# Gene-interleaving patterns of synteny in the *Saccharomyces cerevisiae *genome: are they proof of an ancient genome duplication event?

**DOI:** 10.1186/1745-6150-2-23

**Published:** 2007-09-25

**Authors:** Nicolas Martin, Elizabeth A Ruedi, Richard LeDuc, Feng-Jie Sun, Gustavo Caetano-Anollés

**Affiliations:** 1 Department of Crop Sciences, University of Illinois at Urbana-Champaign, 1101 W Peabody Drive, Urbana, IL 61801, USA

## Abstract

**Background:**

Recent comparative genomic studies claim local syntenic gene-interleaving relationships in *Ashbya gossypii *and *Kluyveromyces waltii *are compelling evidence for an ancient whole-genome duplication event in *Saccharomyces cerevisiae*. We here test, using Hannenhalli-Pevzner rearrangement algorithms that address the multiple genome rearrangement problem, whether syntenic patterns are proof of paleopolyploidization.

**Results:**

We focus on (1) pairwise comparison of gene arrangement sequences in *A. gossypii *and *S. cerevisiae*, (2) reconstruction of gene arrangements ancestral to *A. gossypii*, *S. cerevisiae*, and *K. waltii*, (3) synteny patterns arising within and between lineages, and (4) expected gene orientation of duplicate gene sets. The existence of syntenic patterns between ancestral gene sets and *A. gossypii*, *S. cerevisiae*, and *K. waltii*, and other evidence, suggests that gene-interleaving relationships are the natural consequence of topological rearrangements in chromosomes and that a more gradual scenario of genome evolution involving segmental duplication and recombination constitutes a more parsimonious explanation. Furthermore, phylogenetic trees reconstructed under alternative hypotheses placed the putative whole-genome duplication event after the divergence of the *S. cerevisiae *and *K. waltii *lineages, but in the lineage leading to *K. waltii*. This is clearly incompatible with an ancient genome duplication event in *S. cerevisiae*.

**Conclusion:**

Because the presence of syntenic patterns appears to be a condition that is necessary, but not sufficient, to support the existence of the whole-genome duplication event, our results prompt careful re-evaluation of paleopolyploidization in the yeast lineage and the evolutionary meaning of syntenic patterns.

**Reviewers:**

This article was reviewed by Kenneth H. Wolfe (nominated by Nicolas Galtier), Austin L. Hughes (nominated by Eugene Koonin), Mikhail S. Gelfand, and Mark Gerstein.

## Background

The existence of an ancient whole-genome duplication (WGD) event in *Saccharomyces cerevisiae *[1] has been debated over the past several years. WGD followed by massive gene loss could explain duplicated genes that are interspersed throughout the genome and syntenic relationships with other hemiascomycete yeasts [2-4]. An alternative view is that evolution proceeded gradually through segmental chromosomal duplications that occurred independently, sometimes massively, and were extensively shuffled by recombination [5-10]. Dietrich et al. [11] recently sequenced the *Ashbya gossypii *genome and in a comparative exercise claim their results provide compelling evidence supporting either WGD or a genomic fusion between related species (i.e. a paleopolyploidization) during the early evolution of *S. cerevisiae*. A similar claim comes from the *Kluyveromyces waltii *genome sequence [12]. In both studies, regions of 'double synteny' (DS) were identified in which single genes or groups of genes expressed homology relationships with alternating chromosomal regions of *S. cerevisiae*. This evidence was interpreted as proof of the WGD scenario, and syntenic patterns were considered an indication of the gene order of the most recent common ancestor.

**Figure 1:**
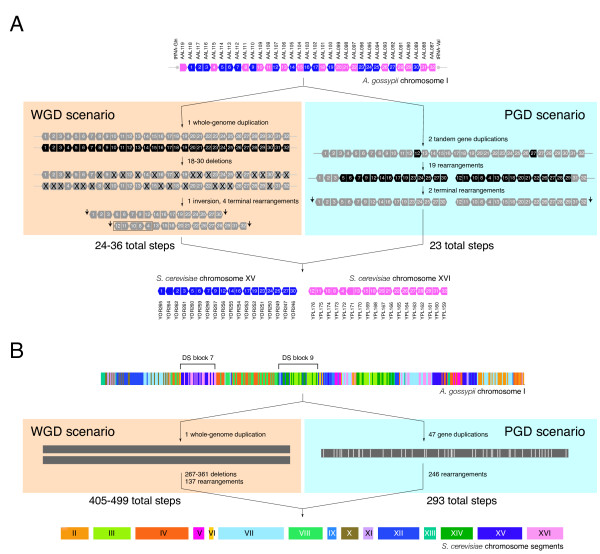
**Comparison of WGD and PGD scenarios in *A. gossypii *DS block 7 (A) and chromosome I (B)**. *A. gossypii *genes and gene segments with homologues in *S. cerevisiae *chromosomes (shown with different colors) are transformed into corresponding *S. cerevisiae *chromosomal segments. When extending results to the entire genome, these segments need to be rearranged into complete *S. cerevisiae *chromosomes. Arrows with numbers in A represent ORFs and their orientation, and are highlighted in black when subject to duplication or rearrangement. Terminal rearrangements are indicated by arrows. Vertical lines in B represent duplications. The absence of *S. cerevisiae *chromosome I homologues of *A. gossypii *chromosome I genes is surprising, given the high levels of rearrangement observed. This suggests biases in synteny that are incompatible with the WGD scenario. The locations of the representative synteny blocks examined are indicated in *A. gossypii *chromosome I.

The *A. gossypii *genome is extremely compact, with seven chromosomes encoding 4,966 protein and RNA molecules [11]. It contains rare gene duplications, only 221 introns, and no transposons of subtelomeric repeats. Although the vast majority of protein-encoding genes (95%) show homology to *S. cerevisiae *genes, only ~10% are gene duplicates in DS patterns. Consequently, the WGD scenario requires that almost an entire gene complement be deleted, and that deletion within regions (termed 'blocks') establishing DS patterns proceed with precision and without leaving massive evidence of relics (gene remnants) or pseudogenes (Fig. 1A). This appears to be unlikely. Recent gene sequence-decay compilations in intergenic regions of *S. cerevisiae *have identified only: (i) 221 disabled open reading frames (ORFs) with middle-sequence frameshifts and premature stop codons (about 3% of the proteome; [13]), and (ii) 278 pseudogenes, including 124 highly degenerated gene remnants termed relics [6], many of which were intermingled with ancestral blocks of duplication [14]. Relics comprising 3% of intergenic regions matched only six over-represented and two under-represented PROSITE motifs (out of 1,319 patterns) in intergenic regions, suggesting pseudomotifs are remnants of very ancient *S. cerevisiae *genes [15]. Moreover, there are 24 triplicated genes (~0.5%) homologous to *A. gossypii *out of 496 duplicated homologues in *S. cerevisiae *[11]. These numbers approach the expected Poisson distribution, suggesting that some of the duplicated regions were subjected to additional duplications in the course of evolution. Furthermore, Kellis et al. [12] also found triplicated regions in *K. waltii*; ~1% of the genome lies in segments that match three or more regions in *S. cerevisiae*.

We believe that the proposal of a massive WGD in the yeast lineage based solely on the evidence of interleaving patterns in sister regions (i.e., DS) is unwarranted and that DS events are more likely a natural topological consequence of a more gradual process of rearrangement within and between chromosomes. Under this evolutionary scenario, gene duplicates originate from partial genome duplication (PGD) events that are local and/or segmental in nature and distribute in time throughout lineages. To test if PGD is more parsimonious than the WGD scenario, we here use Hannenhalli-Pevzner polynomial-time algorithms to transform a numeric representation of one genomic complement into another by rearrangement and to reconstruct ancestral chromosomal complements of genes.

## Results

### Pairwise comparison of gene arrangements in *A. gossypii *and *S. cerevisiae*

We estimated, under the competing PGD and WGD models, the minimum number of evolutionary steps needed to convert an arrangement of genes in *A. gossypii *into the corresponding arrangement in *S. cerevisiae *(see Methods for details). For PGD, we first duplicated genes in tandem and then proceeded to rearrange chromosomal segments. For WGD, we duplicated the entire gene dataset, deleted genes in blocks or individually, and rearranged the resulting segments. The exercise was done using a number of *A. gossypii *syntenic blocks, including DS block 7 (ORFs AAL119W to AAL087C; Fig. 1A) and DS block 9 (ORFs AAL030C to AAR004C; data not shown), both typical examples of DS in chromosome I (*see *Figs. 1 and 2 in [11]). Syntenic relationships related to the entire *A. gossypii *chromosome I were also analyzed (Fig. 1B). In all cases examined, we found PGD was more parsimonious than WGD. A total of 23 steps were needed to transform *A. gossypii *DS block 7 into *S. cerevisiae *chromosome XV and XVI segments under PGD, and 24–36 steps under WGD (Fig. 1A). A total of 30 steps (6 tandem gene duplications and 24 rearrangements) were needed to transform DS block 9 containing the centromere of *A. gossypii *chromosome I into *S. cerevisiae *chromosome III, VII, XII, and XIV segments under PGD, and 37–45 steps (1 segment duplication, 18–26 deletions, and 18 rearrangements) under WGD. Similarly, the transformation of *A. gossypii *chromosome I into 15 *S. cerevisiae *chromosomal segments required 293 steps under PGD or 405–499 steps under WGD (Fig. 1B). Clearly, the difference in parsimony scores between PGD and WGD widens as the sampling becomes more inclusive. Consequently, we expect that a complete analysis of syntenic relationships in the genome of *A. gossypii *will either maintain or broaden the advantage of PGD in parsimony scores. Results therefore underscore the importance of genomic context in comparative analyses.

**Figure 2:**
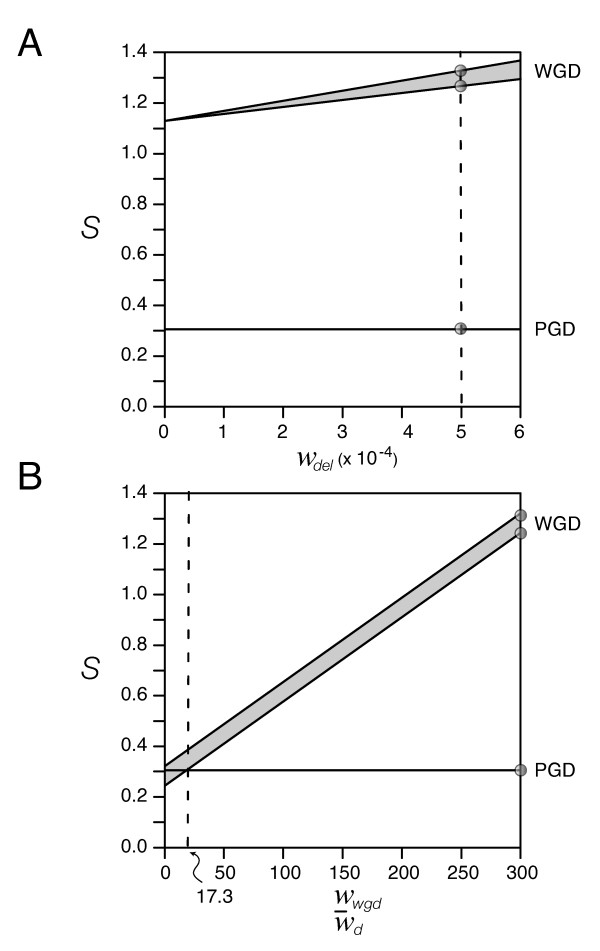
**Effect of weighting schemes on parsimony analysis**. A. Decreasing the weight of deletions has limited effect on parsimony scores supporting the WGD hypothesis. B. Decreasing the weight of genome duplication (relative to the weight of observed gene duplications) renders the WGD scenario possible only if entire genome duplications are considered relatively common events, i.e., occurring at frequencies about an order of magnitude lower than gene duplications. Circles represent reference parsimony scores for the WGD or PGD evolutionary scenarios. WGD scores are given within a range delimited by considering deletions of genes individually or in blocks.

### Impact of weighting schemes

We also used weighting schemes *a posteriori *that consider possible differences in 'effective' rates between duplications, deletions, and rearrangements that occurred since the divergence between *S. cerevisiae *and *A. gossypii*. However, we found that weighting always renders an implausible scenario for WGD when paleopolyploidization events are considered rare (Fig. 2).

Weighted parsimony analyses of *A. gossypii *chromosome I synteny data again suggest WGD is less parsimonious (*S*_WGD _= ~1.2–1.3 weighted steps) than PGD (*S*_PGD _= ~0.3 weighted steps). PGD remains always the preferred scenario (Fig. 2A), even if the probability of a deletion is considered many orders of magnitude greater than that of a rearrangement (as recently argued by Gu and Huang [16]). Only unlikely scenarios will favor WGD. For example, WGD will be more parsimonious than PGD if WGD events occur about an order of magnitude less frequently than gene duplications (i.e., with *w*_*WGD *_<0.057) and only when deletions occur in blocks (Fig. 2B). We believe this is unrealistic. Few gene duplicates exist and large tracts of duplicated segments are not present in yeast to warrant exceedingly high rates of paleopolyploidization.

### Phylogenetic analysis of gene arrangements in *A. gossypii*, *S. cerevisiae*, and *K. waltii *and reconstruction of a common ancestor

Phylogenetic analysis of chromosomal rearrangements in multiple genomes permits the reconstruction of common ancestors and estimation of the number of evolutionary steps that separate ancestors from extant gene complements. These measures were used to test the PGD and WGD hypotheses under more realistic conditions.

**Figure 3:**
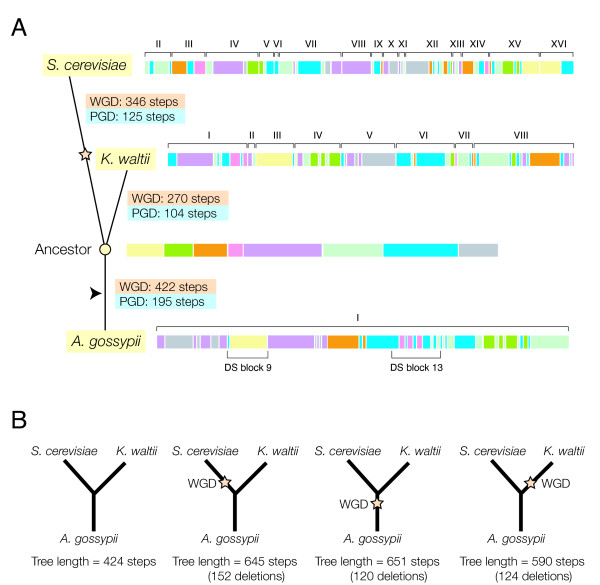
**Phylogenetic analysis of chromosomal rearrangement in *A. gossypii *chromosome I and gene segments with homologues in *S. cerevisiae *and *K. waltii *chromosomes (A) and tree reconstructions under alternative WGD models (B)**. The common ancestral gene arrangement sequence of *A. gossypii, S. cerevisiae*, and *K. waltii (*containing eight segments) was reconstructed (open circle) and genes were transformed into corresponding chromosomal segments (see Fig. 1). Thus, the common ancestral gene sequences embedded in the 8 chromosomal segments of the ancestor were tracked through the corresponding extant sequences of *A. gossypii, S. cerevisiae*, and *K. waltii*. Visual inspection reveals interleaving arrangements of differently colored segments. A detailed analysis of gene order and orientation confirms these interleaving arrangements are synteny patterns. The reconstruction of an ancestor allowed evaluation of which of the competing hypotheses (WGD and PGD) was more parsimonious in lineages leading to extant species. Note that the branches of the trees do not reflect actual lengths, the arrowhead shows the root of the tree based on Kurtzman and Robnett [47], and the star identifies the branch in which the putative WGD occurred [1,12]. The star is also used to indicate the branch defining alternative WGD scenarios.

We first used the set of genes present in *A. gossypii *chromosome I to reconstruct a gene arrangement that was ancestral to arrangements of homologous genes in *A. gossypii, S. cerevisiae*, and *K. waltii *(Fig. 3). Genes that were not shared by the three genomes, including those that were duplicated, had to be removed from further analysis. The reconstructed common ancestor had eight chromosomal segments. Interestingly, gene-interleaving patterns of ancestral chromosomal segments arose naturally and were clearly visible in *A. gossypii *(Fig. 3), suggesting DS patterns arise in the absence of a WGD. These patterns were present in about 70% of *A. gossypii *chromosome I. DS patterns were also visible in *S. cerevisiae *and *K. waltii*. We also calculated the number of steps between the ancestor and the extant gene arrangements under the PGD or WGD hypotheses (Table 1; Fig. 3A). Under the PGD scenario, there were a total of 195, 125, and 104 steps between the ancestor and either *A. gossypii, S. cerevisiae*, or *K. waltii*, respectively. Under the WGD scenario, there were a total of 422, 270, and 346 steps between the common ancestor and either *A. gossypii, S. cerevisiae*, or *K. waltii*, respectively. These results indicate that the PGD scenario is more parsimonious than WGD in all lineages (tree length = 424 steps) (Fig. 3B). However, reconstructed trees under a WGD hypothesis had 651, 645, and 590 minimal steps when the WGD event was placed in the *A. gossypii, S. cerevisiae*, and *K. waltii *lineages, respectively (Fig. 3B). Consequently, the WGD scenario was considerably more likely in the lineage leading to *K. waltii*. There were also two interesting observations: (1) A similar number of steps transformed the ancestor into *S. cerevisiae *and *K. waltii *under WGD and PGD scenarios, respectively, and (2) *S. cerevisiae *and *K. waltii *were evolutionarily closer to the common ancestor than *A. gossypii*. Chromosome I was therefore similarly rearranged in these two lineages. Interestingly, when we use the minimum number of deletions needed to establish gene-interleaving relationships under the WGD hypothesis as an indicator of the complexity of DS patterns, we find that the number of deletions was similar in lineages leading to *S. cerevisiae *and *K. waltii*. This suggests that DS patterns in *K. waltii *are as abundant as those in *S. cerevisiae*.

**Table 1:** Minimum number of evolutionary steps separating *A. gossypii, S. cerevisiae*, and *K. waltii *from the common ancestor under the WGD and PGD scenarios

Events	*A. gossypii*		*K. waltii*		*S. cerevisiae*	
	
	WGD	PGD	WGD	PGD	WGD	PGD
Whole-genome duplication	1	-	1	-	1	-
Gene duplications	-	23	-	17	-	1
Deletions	120	-	124	-	152	-
Reversals	284	140	91	60	114	89
Translocations	2	25	46	27	78	28
Fusions	15	7	8	-	1	7
Total	422	195	270	104	346	125

**Figure 4:**
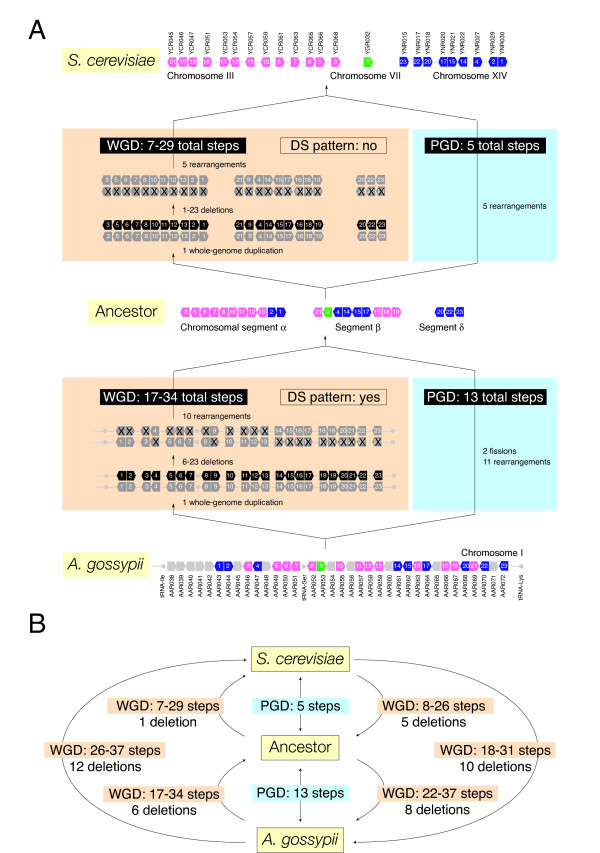
**Phylogenetic analysis of *A. gossypii *DS block 13**. A. Three chromosomal segments homologous to this syntenic region and derived from the phylogenetic reconstruction exercise described in Figure 3 were found to be ancestral to *A. gossypii, S. cerevisiae*, and *K. waltii*. They are labeled with greek letters. The arrangement of genes in *A. gossypii *DS block 13 was transformed into the three ancestral chromosomal segments and these were then transformed into corresponding chromosomal segments in *S. cerevisiae*, invoking both the WGD and PGD evolutionary scenarios. Under WGD, deletions were those that produced the most parsimonious scenario. The minimum number of deletions needed to produce a DS pattern in *S. cerevisiae *was considered to be 1, and not 3, because there was no guarantee that three chromosomal segments were not part of a single chromosome. B. Because under the WGD scenario we first duplicate the entire gene complement and then proceed to delete and rearrange genes, direction of change in lineages is important. The figure shows alternative WGD reconstructions used to select the most parsimonious WGD scenarios shown in A.

We then focused on individual DS blocks, using data from the ancestor of the entire *A. gossypii *chomosome I to reconstruct alternative evolutionary scenarios in lineages leading to extant species. Figure 4 illustrates the approach using *A. gossypii *DS block 13 (AAR069-AAR043) (*see *Fig. 3 of Dietrich et al. 2004). This region defines one of several gene-interleaving relationships in *A. gossypii *describing synteny with ancestral chromosomal segments (pink-and-blue interleaving pattern in Fig. 3). We transformed DS block 13 in *A. gossypii *into the ancestor and then transformed this ancestral gene arrangement into the *S. cerevisiae *counterpart under competing WGD and PGD hypotheses (Fig. 4A), considering only the most parsimonious alternative under each scenario (Fig. 4B). Again, we find PGD was always more parsimonious than WGD. It is noteworthy however, that the DS pattern under WGD arose only in the lineage leading from the ancestor to *A. gossypii *(with 6 minimum deletions) and not in the lineage leading from the ancestor to *S. cerevisiae*. This strongly suggests that the gene-interleaving pattern in DS block 13 did not originate by the proposed WGD after the divergence of *S. cerevisiae *and *K. waltii*, but did so earlier.

**Figure 5:**
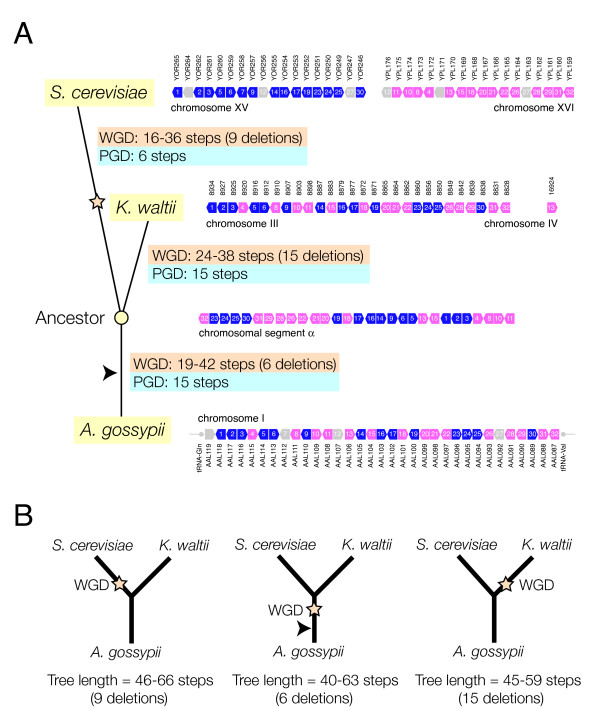
**Phylogenetic analysis of *A. gossypii *DS block 7**. A. The arrangement of genes in the common ancestor of *A. gossypii, S. cerevisiae*, and *K. waltii *that were present in *A. gossypii *chromosome I was reconstructed and used to define the arrangement of genes corresponding to DS block 7. Genes were duplicated and/or rearranged according to the WGD and PGD hypotheses, and the number of evolutionary steps evaluated. Genes in *A. gossypii *and *S. cerevisiae *that were not shared by the three genomes were shaded in gray. For simplicity, they were not illustrated in *K. waltii*. Non-shared and duplicated genes were excluded from analysis. Under WGD, the minimum number of deletions measuring the 'structure' of the DS pattern is also listed. See Figure 3 for the meaning of star and arrowhead. B. Most parsimonious scenarios for a WGD event inferred by tree reconstruction. The length of the tree reconstructed in the absence of WGD was 36 steps.

Figure 5 illustrates how synteny patterns arise within lineages using *A. gossypii *DS block 7 (analyzed in Fig. 1A) as an example. In this analysis, *A. gossypii *genes 7 (AAL112), 12 (AAL107) and 27 (AAL092) had no orthologs in *K. waltii *and had to be removed. The common ancestor of this block fell within a single chromosomal segment, and the number of steps between the ancestor and extant sequences of genes in *S. cerevisiae *and *K. waltii *was calculated under the two alternative hypotheses (Fig. 5A). Under PGD, the number of steps between the common ancestor and *A. gossypii*, *S. cerevisiae*, and *K. waltii *was 15, 6, and 15, respectively. Under WGD, 19–42, 16–36, and 24–38 steps separated the ancestor and extant species, respectively. The minimum number of deletions needed to establish a syntenic pattern in *A. gossypii*, *S. cerevisiae*, and *K. waltii *was 9, 6 and 15, respectively. This indicates that the interleaving patterns of DS block 7 were more structured in *S. cerevisiae *and *K. waltii *than in *A. gossypii*. It is noteworthy however, that the most parsimonious scenario for the existence of a WGD places the event in the *A. gossypii *lineage with 25–48 total steps from *A. gossypii *to *S. cerevisiae *and a minimum tree length of 40 steps (Fig. 5B). In turn, placing the WGD event in the *S. cerevisiae *or *K. waltii *lineage was less parsimonious and required 31–51 steps from *A. gossypii *to *S. cerevisiae *and 39–53 steps from *A. gossypii *to *K. waltii*. The resulting trees had lengths of 45 and 46 minimum steps, respectively. Consequently, lineages leading from the common ancestor of the entire clade (indicated putatively with an arrowhead in Fig. 5) to *S. cerevisiae *should be the largest contributors to the putative WGD event (involving less than 25 evolutionary steps) and corresponding DS pattern (involving less than 6 minimum deletions). Results suggest again that PGD is more likely than WGD and that the most parsimonious explanation of the existence of the WGD event is that it occurred before the divergence of the *S. cerevisiae *and *K. waltii *lineages.

### Orientation of duplicate genes in *S. cerevisiae *with respect to *A. gossypii*

We also analyzed the reading orientation relative to the centromere of the 47 genes present in *A. gossypii *chromosome I that were duplicated in *S. cerevisiae*. It is expected that without rearrangement, duplicated Watson-oriented genes placed at the left of the centromere and Crick-oriented genes placed at the right of the centromere would be read towards the direction of the centromere in *S. cerevisiae*. Left and right are here defined relative to the 5' and 3' ends of the chromosomal sequence, respectively. Conversely, duplicated Crick-oriented genes placed at the left of the centromere and Watson-oriented genes placed at the right of the centromere would be read against the direction of the centromere. Any departures from this expectation would signal the occurrence of gene rearrangement.

When we compared changes in the reading orientation of genes in chromosome I of *A. gossypii*, there were 28 and 19 Watson-oriented and Crick-oriented genes, respectively, and these were duplicated into 45 and 49 Watson and Crick genes in *S. cerevisiae*, respectively. The analyses showed there were a total of 49 changes of gene orientation. We also found that there were 23 and 24 genes placed at the left and right of the centromere for *A. gossypii*, respectively. After the duplication, there were 24 and 70 genes placed at the left and right of the centromere for *S. cerevisiae*, respectively. Therefore, there were 36 changes in the position of the genes with respect to the centromere and genes placed at the left side of the centromere changed their position more often than those at the right.

We also analyzed how gene orientation changed with respect to the centromere. In *A. gossypii*, there were 21 and 26 genes that were read against and toward the centromere, respectively. After gene duplication, there were 49 and 45 genes that were read against and toward the centromere in *S. cerevisiae*, respectively. However, our analysis finds that a total of 51 duplicated genes in *S. cerevisiae *changed their orientation with respect to the centromere.

## Discussion

Gene-interleaving patterns of synteny established between *S. cerevisiae *and *A. gossypii *[11] and between *S. cerevisiae *and *K. waltii *[12] were recently used to support the existence of paleopolyploidization in the yeast lineage. In both of these studies, DS patterns were considered the signature and proof of WGD. However, there were no attempts to evaluate alternative evolutionary models that would explain the DS patterns, and there were no efforts to weight differentially the genome duplication event and the massive loss of duplicated genes necessary to explain the existence of a relatively small number (only 10%) of duplicated genes in the *S. cerevisiae *genome. Using Hannenhalli-Pevzner rearrangement algorithms that address the multiple genome rearrangement problem [17], we here suggest that DS patterns are the natural consequence of topological rearrangements in chromosomes and that a more gradual PGD scenario of genome evolution involving segmental duplication and recombination constitutes a more parsimonious explanation of gene order in the yeast lineage. Four lines of evidence are used here to test if DS patterns are necessary and sufficient conditions for the occurrence of WGD in yeast: (1) pairwise comparison of gene arrangements, (2) reconstruction of ancestral gene arrangements, (3) synteny patterns arising within and between lineages, and (4) expected gene orientation of duplicate gene sets. As with previous analyses that claim DS patterns support the WGD hypothesis [11,12], our phylogenetic studies do not weight the occurrence of rearrangements and other evolutionary processes, since we do not know the rate of these events in the wild and over evolutionary time. Finding these weights represents an extremely challenging task [18].

### Pairwise comparison of gene arrangements

A direct pairwise comparison of gene arrangements from *S. cerevisiae *and *A. gossypii *converted one arrangement into another and produced unweighted parsimony scores that falsified the WGD hypothesis in favor of the gradual evolutionary scenario (Fig. 1). Differential weighting of parsimony scores under more realistic evolutionary assumptions also falsified the WGD scenario (Fig. 2). Decreasing the weight of deletions (relative to rearrangements) had limited effect on parsimony scores supporting the WGD hypothesis. Similarly, decreasing the weight of the genome duplication supported the WGD scenario only at unusually high genome duplication frequencies (an order of magnitude lower than gene duplications).

It could be argued that strongly penalizing the WGD event can bias the analysis. However, there are reasons to believe yeast paleoploidization is a rare event [18]. Polyploid lines exhibit high levels of chromosome instability [19,20] while segmental duplications are rare, but occur frequently over evolutionary time [9,18]. In fact, according to the WGD hypothesis, paleopolyploidization occurred once in the history of hemiascomycete fungi [10], possibly after the divergence of the *S. cerevisiae *and *K. waltii *lineages [1,12]. The fact that a small fraction of genes in yeast are duplicated and that there are few relics or pseudogenes that were left behind [12-15] suggests polyploidization and gene deletion events occur at low evolutionary frequencies in yeast. The existence of polyploid strains of yeast resulting from defects in mitosis or interspecific mating need not detract from this argument because we have limited understanding of the long-term stability of these lines. Gene deletion should also be regarded a slow process. In many cases, more than half of gene pairs are retained over tens of millions of years following genomic doubling [21], though instantaneous rates of deletion could be higher immediately following events of duplication.

In our studies, unweighted parsimony analysis should be considered a more conservative 'falsification' tool that eliminates assumptions introduced by weighting schemes, especially because the estimation of weights from incidence values may be misleading. Estimating actual rates of duplication, recombination, and deletion during the 100 Myr of evolution encompassing the putative WGD event constitutes a difficult proposition, especially because rates vary with time. For example, gene duplicates arise at high rates in yeast, about 0.0083 per gene per Myr, but the process varies with the level of sequence divergence and is counteracted by the relatively short half-life of individual genes (~4 Myr) due to active gene silencing and loss [22,23].

It could be argued that conservation of gene order in DS patterns, the cornerstone of the WGD hypothesis, cannot be explained by the PGD scenario. For example, rearranging *A. gossypii *chromosomes by their very nature perturbs the original gene order evident in the genome decreasing considerably the *a priori *probability of obtaining similar patterns by rearrangement in other genomes (e.g., in *S. cerevisiae)*. Following this argument, DS patterns under the PGD hypothesis would ultimately arise as a consequence of a highly fortuitous and unlikely sequence of rearrangement events. This frequentist argument is fallacious under the light of evolution, and ultimately critiques the unrealistic and unidimensional nature of pairwise gene arrangement analysis, in which one extant genome transforms into another. Gene arrangements arise from a common ancestor, and a valid comparison of contemporary genomic patterns has only value if supported by phylogenetic reconstruction of ancestral gene arrangements. For example, the number of shared traits in lineages increases with decreases in taxonomic distance. Extensive sharing of traits in closely related lineages could be erroneously interpreted as a highly fortuitous and unlikely sequence of events. However, traits arise by common descent with modification, with changes accumulating gradually according to some model (e.g., WGD or PGD scenarios) and resulting in evolutionary patterns adequately revealed by modern phylogenetic analysis. In this regard, gene arrangements are not different from any other trait, whether these are molecular, biochemical, or morphological. We here contend that DS patterns arise as the result of gradual change in evolutionary lineages and should be regarded as *shared and derived features *that were inherited from an ancestor and not as fortuitous and unlikely events.

### Phylogenetic analysis of multiple genome arrangements

The pairwise comparison of gene arrangements previously used is grounded in phylogenetic analysis but does not assume the existence of a phylogenetic tree. To compare evolutionary hypotheses under more realistic conditions, we used the multiple genome rearrangement algorithm in GRIMM to generate a phylogenetic tree and reconstruct ancestors (Fig. 3). The algorithm evaluates, for example, all possible chromosomal reversals for a set of genome complements, selecting those 'good' reversals that bring all genomes closer to the ancestral genome and iterating the procedure until all genomes are transformed into it [17]. The reversal distance should be considered a good approximation of the true distance for many biologically relevant cases, and is currently widely used in the analyses of genome rearrangements [24]. GRIMM was recently used to reconstruct an ancestral murid genome using the human, mouse, and rat genomes [25] and to analyze mammalian chromosome evolution in a multi-species phylogenomic comparison [26].

We attempted a rearrangement analysis of multiple genomes but this was not possible because of the high levels of recombination present in hemiascomycete yeasts (N. Martin, unpublished data). Despite computational limitations, we built a phylogeny describing the evolution of genes present in *A. gossypii *chromosome I along the *S. cerevisiae*, *K. waltii*, and *A. gossypii *lineages, reconstructing a gene arrangement that was present in the common ancestor to these species (Fig. 3). Transforming the arrangement of genes in the ancestor into arrangements in extant species under competing PGD and WGD hypotheses again falsified the paleopolyploidization event (Table 1; Fig. 3). As with pairwise analysis, PGD was always more parsimonious than the WGD scenario.

Interestingly, the minimal number of deletions needed to establish gene-interleaving patterns with the ancestor under WGD was comparable in the lineages leading to *S. cerevisiae *and *K. waltii*. This suggests these two genomes had DS patterns that were similarly structured. In fact, interleaving patterns of ancestral chromosomal segments were clearly visible in contiguous fragments of all three extant genomes analyzed, visualized as interleaving arrangements of differently colored segments in Figure 3. In particular, DS patterns could be detected in about 70% of chromosomal regions of chromosome I in *A. gossypii*. Consequently, gene-interleaving patterns cannot be considered an exclusive property of lineages that have undergone a WGD.

### Synteny patterns arising within and among lineages

According to the WGD hypothesis, paleopolyploidization occurred after the divergence of the *S. cerevisiae *and *K. waltii *lineages [1,12]. In fact, DS patterns arising from the comparisons of *S. cerevisiae *and *A. gossypii *[11] and *S. cerevisiae *and *K. waltii *[12] genomes were given as irrefutable proof of paleopolyploidization. Consequently, the WGD event had to occur in the lineage leading to *S. cerevisiae *and after the divergence of *K. waltii *(marked with a star in Fig. 3) if DS patterns depicted paleopolyploidization appropriately. However, phylogenetic analyses of gene orthologues duplicated in *S. cerevisiae *suggest genes duplicated and/or specialized before the *S. cerevisiae *and *K. waltii *lineages diverged from each other [27]. This is clearly incompatible with the WGD hypothesis.

The presence of gene-interleaving patterns in the genomes of the three fungi examined in this study is also incompatible with the WGD hypothesis and suggests patterns of synteny arise naturally as a result of duplication and rearrangement. To visualize the evolution of individual syntenic patterns, we used the reconstructed ancestral gene arrangements of *A. gossypii *chromosome I to study individual DS blocks defined by Dietrich et al. [11]. In these analyses, small groups of genes present in defined order in the ancestral complement produced DS patterns between extant genomic regions in relatively few steps under competing WGD hypotheses. This allowed placement of the WGD event in alternative branches of the reconstructed phylogenetic tree, evaluating the placement that is the most parsimonious.

DS block 13 is one of several synteny blocks in chromosome I of *A. gossypii *(*see *Fig. 3 of Dietrich et al. [11]). This block represents fundamentally an interleaving pattern of *S. cerevisiae *chromosome II and XIV segments, though it also involves chromosome VII. We chose this DS pattern because it coincides with an interleaving arrangement of ancestral chromosomal segments in *A. gossypii *that is illustrated with alternating pink-and-blue colored segments in Figure 3. Explaining gene order in the common ancestor under the WGD hypothesis showed the DS pattern arose in the lineage leading to *A. gossypii*, before, but not after the divergence of *S. cerevisiae *and *K. waltii *(Fig. 4). This single example shows not all DS patterns in blocks reported by Dietrich et al. [11] were involved in the putative WGD.

We also analyzed DS block 7, the example DS pattern used by Dietrich et al. [11] to illustrate the basis of the genome duplication model (Fig. 5). Transformation of the ancestor into extant gene arrangements under competing hypotheses revealed how DS patterns developed in the making of this syntenic block. Under WGD, interleaving patterns were less structured but were generated more parsimoniously in the *A. gossypii *and *S. cerevisiae *lineages than in the *K. waltii *lineage (Fig. 5A). In fact, the most parsimonious explanation of the WGD event proposed to have occurred in the *S. cerevisiae *lineage [1] places it in the lineage that originates in the common ancestor of the clade (arrowhead) prior to the divergence of the *S. cerevisiae *and *K. waltii *ancestors (Fig. 5B).

Since many syntenic patterns mapping onto *A. gossypii *chromosome I could be explained by interleaving patterns occurring early, prior to the divergence of *S. cerevisiae *and *K. waltii*, we extended our comparative approach to the entire *A. gossypii *chromosome I (Figure 3). Surprisingly, alternative tree reconstructions did not place the WGD event in the lineage leading to *S. cerevisiae *as would have been expected. Instead, the most parsimonious explanation for a genome duplication event was that it occurred after the divergence of the *S. cerevisiae *and *K. waltii *lineages, but in the lineage leading to *K. waltii*. This is clearly incompatible with the WGD scenario proposed when comparing syntenic regions in the genomes of *S. cerevisiae *and *A. gossypii *[11], and *S. cerevisiae *and *K. waltii *[12].

Note than in these comparative experiments, it is the WGD scenario the one that was unable to recover the paleopolyploidization event in the right branch of the tree. Arguments of differential weighting of steps cannot be brought to question these results, which are ultimately based on the syntenic relationships and patterns that have been used to support the WGD hypothesis in pairwise analyses [11,12]. We focus here exclusively on the genomes that were used to postulate DS patterns as irrefutable proof of the ancient duplication event in yeast. However, our comparative analyses could be extended to other genomes that speciated presumably after the WGD event, such as *S. castelli *[28].

Overall results therefore confirm that the existence of DS patterns is a condition that is *necessary but not sufficient *to support the existence of paleopolyploidization. Therefore, caution should be exercised when using DS patterns to support WGD claims [11,12]. The fact that about 90% of the *S. cerevisiae *genome is involved in establishing syntenic relationships with closely related fungi, such as *A. gossypii*, cannot be used as proof of ancient tetraploidy occurring after the divergence of *K. waltii *and *A. gossypii *[11,12], because DS events appear to occur earlier in the yeast lineage and in other lineages believed to be free of paleopolyploidization events. DS patterns are therefore likely to be the direct consequence of chromosomal rearrangement and segmental duplications and to emerge naturally under the more parsimonious PGD scenario. In this gradual process of change, segmental duplications are expected to distribute along branches in the tree.

Our results also show that the generation of interleaving patterns of synteny under a WGD scenario is a complex process in which chromosomal rearrangement plays an important role. We still need improved models of rearrangement that incorporate segmental duplication of extensive regions of the genome. Despite this limitation, phylogenetic reconstruction experiments here described falsify the WGD hypothesis as such in favor of a more gradual evolutionary scenario.

### Gene orientation of duplicate gene complements

Wolfe and Shields [1] studied the orientation of duplicated regions (blocks) with respect to the centromere in *S. cerevisiae*. These regions were identified by amino acid sequence similarity. Under a WGD scenario, the expectation was that block orientation would be conserved if blocks were formed by reciprocal translocations among duplicate chromosomes. Under a competing PGD scenario, independent rearrangement of blocks would result in random orientations. Analyses of *S. cerevisiae *duplicate regions revealed that 50 out of 55 regions did not change in orientation and this was given as evidence in support of a WGD hypothesis driven by "tetraploidy and translocation" [1]. Following this line of reasoning, we analyzed the orientation relative to the centromere of the 47 genes in chromosome I of *A. gossypii *that were duplicated in *S. cerevisiae*. In our case, gene orientation defines the orientation of a duplicated region that was not rearranged during the evolutionary time frame considered. There were 49 changes of gene orientation and 36 changes in the position of genes with respect to the centromere. As a result, there were 51 changes in gene orientation with respect to the centromere. These changes were substantial (almost half of duplicates) and support the existence of independent chromosomal rearrangements compatible with the PGD scenario.

## Conclusion

It is true that many diploid species actually represent paleopolyploids. Polyploidy may have occurred in the lineage of at least 70% of angiosperms [29], and it is clearly a revolutionary and ongoing process in the grasses [30]. We recently traced the evolution of genome size in lineages of the grass family and found several instances of genome size increase, some quite notable, which could be explained by paleopolyploidization [31]. Plant paleopolyploidy is supported by genomic and phylogenetic analyses [32-34] and may have had an important role in the origin and evolution of angiosperms [35,36]. However, the controversial proposal that genome evolution is mainly driven by WGD [37] and is widespread in vertebrates and fungi has been intensely debated [5,38-40]. Hughes et al. [41] used parsimony criteria and phylogenetic analyses to falsify the existence of ancient genome duplication events that would structure *Hox*-bearing human chromosomes. The validity of using 'parsimony tests' like these to falsify the WGD hypothesis has been recently questioned [16,40]. However, statistical considerations failed to disprove the validity of the test and there was no attempt to measure the actual rearrangement process. This is necessary in order to unravel the actual meaning of synteny patterns.

Untangling the elements of gene order embedded in a genome represents a critical problem for comparative genomics [42]. The apparent simplicity of the WGD scenario in its ability to explain syntenic relationships in pairwise comparison is attractive, but can be misleading. Our studies show that a PGD alternative that involves only tandem gene duplications and rearrangements is consistently more parsimonious and explains the order and directionality of genes in fungal chromosomes.

The WGD scenario seems poorly compatible with analyses of contemporary polyploids that show increased ploidy is an inherently unstable state. For example, in recent experiments, autotetraploids of *S. cerevisiae *had elevated rates of chromosome instability and died rapidly in stationary phase [20]. In contrast, the PGD scenario certainly matches the dramatic ability of *S. cerevisiae *to increase the frequency of chromosomal rearrangements under environmental stress, a feature that could facilitate sympatric speciation in starving populations (E. Kroll, personal communication). It is also compatible with mitotic and meiotic stability of interchromosomal duplications and direct tandem duplications [9,43]. In contrast, in these studies, large duplications carried by a supernumerary chromosome were highly unstable.

While more parsimonious, the PGD scenario here proposed does not consider evolutionary contributions from segmental duplications, events known to be common in hemiascomycete yeasts [7-10]. We believe the associated loss of gene duplicates generated from the successive accumulation of segmental duplications should be inferred from relics left behind by gene decay [13-15] and used in future evolutionary models.

To conclude, our results disprove the concept that the existence of DS patterns constitutes compelling evidence of paleopolyploidization in the *S. cerevisiae *lineage [11,12]. A more gradual evolutionary scenario explains DS patterns more parsimoniously. Under Popperian falsification criteria, our results prompt to restate the null hypothesis that paleopolyploidization did not occur in yeasts. However, computational limitations restrict our analyses to a single chromosome in *A. gossypii*. This also limits the falsification test because the corresponding homologous regions are scattered into many non-consecutive synteny blocks in virtually all chromosomes in *S. cerevisiae *and *K. waltii*. Extension of these studies to entire genomes, the use of experimentally defined weights for gene duplication, deletion, and WGD events, and the use of rearrangement models that allow inclusion of small and large duplication and deletion events should refine and extend our conclusions.

## Methods

We analyzed rearrangements of gene order from signed gene data derived directly from genome annotation of the *A. gossypii*, *K. waltii*, and *S. cerevisiae *genomes [11,12] using the Genome Rearrangements In Man and Mouse (GRIMM) web server [44]. A genome was represented as a signed permutation of gene numbers 1, 2, 3, ..., *n *spreading over *m *chromosomes or genome segments, with signs '+' and '-' indicating the two possible orientations of a gene (hereafter termed Watson and Crick, respectively). GRIMM addresses the *pairwise genome rearrangement problem *using Hannenhalli-Pevzner algorithms that find the minimum number of rearrangement operations [e.g., inversions (reversals), translocations, fissions, and fusions] necessary to transform one genome into another, when genomes contain the same gene set and each gene appears exactly once in a genome. The rearrangement algorithms have been recently used to study genome rearrangements in the very difficult *Campanulaceae *cpDNA dataset [17] and breakpoint reuse in mammalian evolution [26,45,46]. GRIMM does not separate and bring genes back together in artifactual patterns. GRIMM tries to explain rearrangements with a minimum number of rearrangement operations. In short segments (e.g., a single synteny block; Fig. 1A), the operation is restricted and a pattern of shuffling may appear evident. However, when examining detailed rearrangement operations in an entire chromosome, genes change their positions in large groups, and not as individual localized jumping events.

In our study, we combined rearrangement operations with duplication and deletion events, we gave all of them initially the same weight, and we used combined estimates of the minimum number of evolutionary steps (changes) as parsimony scores in support of the PGD or WDG scenarios. Under PGD, gene duplications were forced to occur in tandem, and duplicates were given a new gene number. Out of all possible duplicate gene combinations in DS blocks, optimal (most parsimonious) evolutionary scenarios were those that required the least number of steps. These parsimony scores should be considered conservative estimates. For example, when conducting pairwise analysis of syntenic relationships in *A. gossypii *chromosome I, the 47 gene duplicates were randomly assigned to alternative *S. cerevisiae *segments without attempting optimization. Since some of the 2^47 ^possible combinations are expected to be more parsimonious than those arising by random assignment, parsimony scores under PGD should be regarded as conservative upper bounds. Under WGD, we counted the deletion of genes that follow the WGD event either in blocks or individually, prior to rearrangement. Consequently, parsimony scores were given as a range with an expectation that the most realistic scenario will match some central tendency. Note that alternative PGD and WGD scenarios can be proposed in which duplications and deletions are allowed to occur at different times during rearrangement. Computation of parsimony scores for these alternative scenarios is complex. However, scores should not differ significantly from those reported, especially if segmental duplications and deletions involve sets of few genes.

We also weighted the number of rearrangement (*r*), gene duplication (*d*), genome duplication (*wgd*), and deletion (*del*) events *a posteriori*, to account for possible differences in the effective rates of these evolutionary processes. Total weighted parsimony scores (*S*) representing the WGD (*S*_WGD_) or PGD (*S*_PGD_) evolutionary scenarios were calculated according to the general formula

*S *= *w*_*r*_*s*_*r *_+ *w*_*d*_*s*_*d *_+ *w*_*wgd*_*s*_*wgd *_+ *w*_*del*_*s*_*del*_

with *s *representing the number of events and *w *the relative weights associated with them. Weights were calculated from the actual genomic incidence of these events. According to the WGD model, polyploidization occurred once in the history of hemiascomycete fungi [10] and within the 100 million year (Myr) period since the divergence between *S. cerevisiae *and *A. gossypii*. During this period, about 1,000 rearrangements and 2,000–2,500 deletions had to accompany the WGD event in order to explain synteny data (Fig. 1B). Alternatively, the PGD scenario suggests that about 300 gene duplications and 1,700 rearrangements occurred during the same period. Weights for rearrangements (*w*_*r *_= 0.0005–0.001), gene duplications (*w*_*d *_= 0.003), entire genome duplications (*w*_*wgd *_= 1), and deletions (*w*_*del *_= 0.0004–0.0005) were used to estimate reference *S*_WGD _and *S*_PGD _scores, and study how individual weights affected parsimony scores.

Comparing more than two gene arrangement sequences (referred here as 'gene arrangements') allows reconstruction of a common ancestral arrangement (referred here as the 'common ancestor') to the gene arrangements analyzed. Reconstruction requires that the gene complement be shared by all genomes considered. Under competing PGD and WGD models, we determined the minimum number of steps needed to convert the arrangement of shared genes in *A. gossypii *chromosome I (307 common genes) of the ancestor of *A. gossypii, K. waltii*, and *S. cerevisiae *into the corresponding chromosomal arrangement in extant species. To achieve this goal, the ancestral arrangement common to *A. gossypii*, *K. waltii*, and *S. cerevisiae *gene complements was inferred by minimizing the number of translocations, reversals, fissions, and fusions. The number of chromosomal rearrangements needed to generate the ancestor and the genes that were duplicated defined the number of evolutionary steps separating the ancestor and the three extant sequences under PGD. We also estimated the number of steps under the WGD scenario for lineages leading to *S. cerevisiae *[11] and *K. waltii *[12]. Initially we created an entire genome duplication that was then followed by massive deletions. We assumed that for every change in order in an extant gene arrangement, a deletion occurred following the WDG event, generating a gene-interleaving pattern. Consequently, the ancestral arrangement of genes was generally transformed into two gene arrangements that complemented each other. By the same token, the ancestor was transformed in GRIMM to extant *S. cerevisiae *and *K. waltii *chromosomal arrangements. Thus, the parsimony score under WGD included the global duplication event, subsequent deletions, and rearrangements, excluding the number of gene duplications. In these analyses, the phylogenetic tree that was reconstructed was rooted in the branch leading to *A. gossypii *according to Kurtzman and Robnett [47].

## Competing interests

The author(s) declare that they have no competing interests.

## Authors' contributions

This study began as a class project in CPSC 569, a course taught by GCA at the University of Illinois in spring 2004. NM acquired genomic data, performed phylogenetic analyses, and analyzed results. EAR, RL, and FJS were mostly involved in pairwise analyses and contributed equally to this work. GCA guided research and wrote the paper. GCA and NM answered reviewer's comments and performed further analyses. All authors read and approved the final manuscript.

## Reviewer's commentss

### Reviewer's report 1

Kenneth H. Wolfe, University of Dublin, Trinity College, Dublin 2, Ireland (nominated by Nicolas Galtier, CNRS-Université Montpellier II, France). 21 May 2007

Martin et al. are mistaken. The answer to the question they pose in their title is "Yes". There are several different lines of evidence that all support the WGD hypothesis, and the interleaving pattern is one such line of evidence. I suppose that one could make the philosophical argument that in evolution it is impossible to absolutely prove anything, but the evidence for WGD is so strong that I think that any reasonable scientist would consider it proven at this stage.

Martin et al. place too much faith in the Hannenhalli-Pevzner algorithm. They ignore the fact that, even though their PGD model can explain the observed data using fewer rearrangement steps than the WGD model, the PGD model requires an exceedingly unrealistic pattern of rearrangements to happen. In order for their model to produce the observed pattern of double conserved synteny (DCS) interleaving, a bizarre series of nested inversions of pieces of DNA of progressively increasing size must have occurred, as I describe in Comment 1 below. There is no known evolutionary mechanism that can produce such a pattern, and in my opinion this makes the PGD model untenable, regardless of the results of the Hannenhalli-Pevzner algorithm. Parsimony is not just a question of how many steps are required, but also whether those steps are plausible events.

#### Author's response

**Figure 6:**
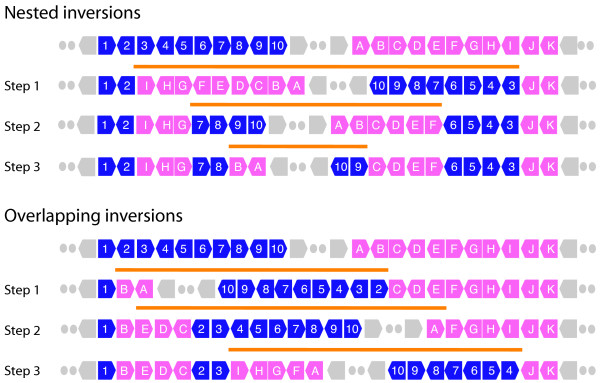
**A series of nested or overlapping inversions produce DS patterns at ends of the rearranged segments**. Yellow lines between steps indicate the chromosomal segments that are inverted between rearrangement steps. Note that the complexity of resulting DS patterns is enhanced if genes highlighted in pink or blue result from previous translocations between chromosomes, or if the inversion process is combined with fissions, fusions and translocations. Moreover, the timing of rearrangement and the lineage in which the rearrangement occurs will further delimit the DS patterns produced. Ultimately, DS patterns arise from a comparative exercise that is set by the user.

*We disagree with Dr. Wolfe's statements. As we will elaborate below, DS patterns of rearrangements under the PGD model are not "unrealistic" or "bizarre". The generation of these patterns under the PGD hypothesis does not require "a complex series of events to happen in an orchestrated manner" as the reviewer suggests. They can be considered the natural consequence of the chromosomal rearrangement process, very much as genome duplication and selective deletion of genes can be considered the engines of the WGD model. Even in cases that involve nested inversions in hot spots of rearrangement, they can be explained by known biological phenomena (see below). In fact, two or more simple steps of nested or overlapping inversions (reversals) will produce DS patterns at two ends of the segments that are being rearranged (*Fig. 6*). This process is clearly more parsimonious and plausible than a rare WGD and many targeted deletions*.

*In our study, we use algorithmic operations (steps) of chromosomal rearrangement, duplication and deletion to compare the WGD and PGD hypotheses or to establish in which lineage the WGD occurred. All operations should be considered plausible events, as they all represent outcomes of known biological phenomena (e.g. recombination, mutation processes, etc). We use these operations to challenge the concept that DS patterns constitute irrefutable proof of the WGD model *[11,12]*by using Ockham's razor ('Pluralitas non est ponenda sine neccesitate'), a principle of preferring simple explanations in hypotheses to complex ones. This principle is fundamental to scientific inquiry. Because the rearrangement process is a critical and complex problem *[42], *we use one of the most advanced algorithms known to date to reconstruct rearrangement operations in genome evolution. The algorithm has been used successfully for example to study genome rearrangement in mammals *[26]. *The issue is not to "place faith in the Hannenhalli-Pevzner algorithm", but to test the validity of WGD versus a null hypothesis of no WGD with this modern bioinformatic tool. It turns out that our analysis falsifies the WGD hypothesis and that under this light, the evidence of paleopolyploidization in yeast may not be so strong as Dr. Wolfe contends. It now behooves WGD supporters to find an improved rearrangement algorithm that does not falsify their model*.

#### Comment 1. The PGD model requires a highly unlikely series of nested inversion events to have occurred in each interleaved region

Let us consider the genomic region used in Martin et al's Figure 1A, corresponding to *Ashbya gossypii *chromosome I genes AAL118C to AAL087C. Additional File 1 shows a multispecies view of this region using the Yeast Gene Order Browser [48]. My explanation of this pattern is that an ancestor of *S. cerevisiae*, *C. glabrata*, *S. castellii *and *K. polysporus *had a gene order essentially identical to what is currently seen in *A. gossypii*, *K. waltii *and *K. lactis*. After WGD in the common ancestor of the first four species, many genes were deleted. In *S. cerevisiae *this left the current paired region between chromosomes XV and XVI. The interleaving pattern was later disrupted slightly by a subsequent inversion of the region between YPL172C and YPL176C on chromosome XVI, as marked in Additional File 1. The left part of Martin et al's Figure 1A describes this scenario accurately, with 24–36 steps (depending on the size of the deletions), though I note that the 4 "terminal rearrangements" are actually reciprocal translocations.

The PGD scenario for the same region is summarized in Martin et al's Figure 1B. They say that it is more parsimonious because the total number of steps is only 23. But the steps are very strange. In Additional File 2 I show the details of the 19 rearrangement steps required in this panel (reconstructed using the GRIMM server, the same method used by Martin et al.). The 19 steps are a series of nested inversions, centered on gene 12 and getting progressively larger. They make a series of flip-flop sorting movements that gradually moves all the red genes to the left, and all the green genes to the right. Look at how gene 18 ends up beside gene 20. At step 7→8, the link between genes 18 and 19 is broken by inverting an eight-gene region (shown by the yellow bar). At the next step, step 8→9, a slightly larger nine-gene region that spans and extends from the original eight, undergoes reinversion so that gene 18 is placed beside gene 20, and gene 19 ends up in the growing red area on the left. In total, in the course of the 19 steps required under the PGD model, gene 12 undergoes inversion 19 times; gene 14 is inverted 17 times, gene 16 is inverted 15 times, and so on (see Additional File 3). I do not know of any evolutionary mechanism that can result in a nested series of inversions of progressively increasing size like this. If one made 19 random inversions in this region, it would be exceedingly unlikely that they would form a nested pattern like this. If the PGD model is correct, it implies that there were 55 (or more) central points, such as gene 12 above, that were continuously inverting and re-inverting, each time carrying a slightly larger region around them, in order to form the 55 interleaved regions that we now see [1]. Moreover, each of these whirlpools of inversion managed to perform the flip-flip sorting process without crashing into the next whirlpool further along the chromosome; otherwise we would see triple- or quadruple-interleaving patterns, but we do not.

Hence I consider the PGD model unparsimonious. It requires a complex series of events to happen in an orchestrated manner, with no obvious mechanism, to produce the synteny relationship between *A. gossypii *and *S. cerevisiae *that we now see. In contrast, the WGD model requires only one unlikely event (the WGD itself) followed by a lot of very simple events (gene deletions, and reciprocal translocations at random genomic sites).

On Discussion paragraph 4 (page 8) Martin et al. admit that it could be argued (as I do) that under the PGD model the double-synteny patterns "would ultimately arise as a consequence of a highly fortuitous and unlikely sequence of rearrangements". I do not understand the basis on which they then reject this argument as "frequentist" and fallacious, but they do say that "valid comparisons need to reconstruct the ancestral gene arrangement first". I show in comment 2 below that they have failed to reconstruct the ancestral arrangement correctly.

#### Author's response

**Figure 7:**
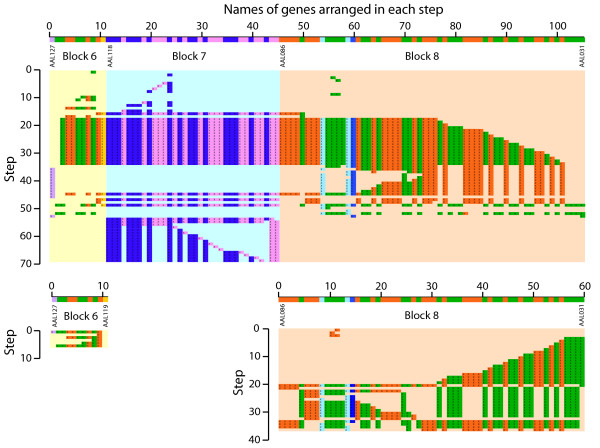
**Summary of genes rearranged at each step in DS block 6, 7 and 8 or individually in DS block 6 and DS block 8 under the PGD model**. As in Additional File 3, we tallied genes that are involved in rearrangement steps of reversals (inversions), transpositions, fissions and fusions and belong to DS blocks 6, 7 and 8 (105 genes, 69 steps), DS block 6 (8 genes, 6 steps) and DS block 8 (60 genes, 37 steps).

**Figure 8:**
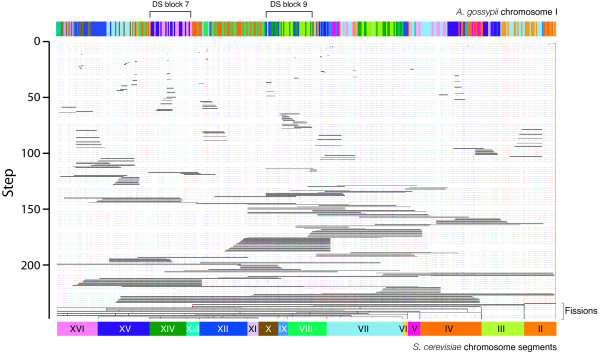
**The 246 rearrangement steps necessary to transform *A. gossypii *chromosome I into chromosomal segments that are homologous in *S. cerevisiae *according to the PGD scenario**. The diagram was obtained directly from GRIMM. Black horizontal bars show groups of genes rearranged at each step and are analogous to the yellow bars described in Additional File 2 for DS block 7. Vertical colored lines indicate chromosome segment ends ("caps") and show effect of fusions and fissions. Chromosomal segments of source and destination genomes are colored as in Figure 1. Note that rearrangements involving the largest number of genes (bottom of diagram) are roughly centered on the centromere (located at the right hand side of DS block 9).

*Dr. Wolfe points out appropriately that under PGD and during rearrangement of A. gossypii DS block 7 (encompassing A. gossypii genes AAL188 to AAL087) a series of nested inversions centered on gene AAL107 (gene 12 of *Additional File 3*) occur. For accuracy, we would like to mention that *Additional files 2*and *3*describe several different rearrangement operations besides inversions (reversals). In fact, the optimal transformation of DS block 7 into S. cerevisiae homolog regions involves 6 translocations and one fission, besides the first 12 reversals, and these operations are not all centered on AAL107. Dr. Wolfe then critiques the rearrangement patterns of nested inversions as being unnatural, stating there is no known evolutionary mechanism that can produce them. This is incorrect. Nested inversions are common, have functional roles in programmed gene rearrangement processes in bacteria *[49], *and occur even in large tracts for example around bacterial origins of replication *[50]*or plant chromosomal arms *[51]*The existence of 'hot spots' of rearrangements with one end in 'fragile breakage' sites and another in random locations may be common in mammalian genomes and the result of long regulatory regions and inhomogeneity of gene distribution *[46,52]. *Besides mammals *[26], *the existence of fragile sites has been confirmed in other genomes, including Drosophila *[53], *and our knowledge of why recombination hot spots occur preferentially in certain genomic regions is expanding and suggest common mechanisms for their formation and function in eukaryotes *[54]. *In particular, hot spots in yeast are not distributed randomly and can be associated with transcriptionally active regions, nucleosome excluding sequences, and GC rich chromosomal regions *[54]. *Consequently, inversion "whirlpools" captured by the rearrangement algorithm at global scale may represent natural phenomena in yeast. Dr. Wolfe also questions the unlikely nature of having inversion whirlpools occurring in each DS block that could collide with each other to form triple- or quadruple-interleaving patterns. This concern is again unfounded. As we state in Methods, when restricting rearrangement analysis to chromosomal segments, patterns of shuffling (the whirlpools) may appear evident, but such patterns vanish or distribute as the analysis is extended to the entire chromosome. We thank Dr. Wolfe for bringing this subject to discussion because it induces clarification of the importance of genomic context. It is unnatural to perform rearrangement operations in a DS block, because the algorithm fails to use other genomic regions in more parsimonious rearrangement scenarios. Consequently, DS block-specific whirlpools are generally replaced by more global rearrangement patterns as one encompasses more genomic sequence around the DS block (culminating with the chromosome containing the block and then the entire genome). To illustrate this point, we analyzed DS block 7 in conjunction with neighboring blocks 6 and 8 (encompassing A. gossypii genes AAL127 to AAL031), together or in isolation (*Fig. 7*). Original whirlpool patterns of individual DS blocks disappear and are replaced by more global rearrangement processes that involve genes in all three blocks. Rearrangement patterns for genes in A. gossypii chromosome I become even more global (*Fig. 8*). In conclusion, whirlpools do not collide with each other and the PGD scenario, very much as the WGD model, involves many simple events that are plausible. In fact, two or more inversion steps can generate one or two DS blocks in regions that are terminal to the rearranged fragments (*Fig. 6*)*.

### Comment 2. The ancestral gene order inferred by Martin et al. does not make sense

In Figures 3, 4, 5, Martin et al. consider the evolution of gene orders in *S. cerevisiae*, *A. gossypii *and *K. waltii *from their common ancestor. To do this, they tried to infer the gene order that existed in the common ancestor, but they did not do this correctly. Consider their Figure 5, which shows the same region that I have shown in Additional File 1. In this region the *A. gossypii *and *K. waltii *gene orders are essentially identical (see Additional File 1); they are colinear (the only differences between them are the absence of a homolog of gene YOR264W in *A. gossypii*, and the absence of a homolog of YOR258W in *K. waltii*). Therefore, parsimony says that the common ancestor of *A. gossypii *and *K. waltii *had virtually the same gene order as each of these species has today. Indeed, the same gene order is also seen in *K. lactis *(Additional File 1). But Martin et al's Figure 5 shows an ancestor (chromosomal segment alpha) that is 15 steps different from each of *K. waltii *and *A. gossypii *under a PGD model. Their scenario implies that an identical series of 15 rearrangement steps happened convergently in both *K. waltii *and *A. gossypii *after they diverged from their common ancestor. This scenario is so unrealistic that I cannot believe that any of the results they derive from this ancestral order are accurate.

#### Author's response

*The argument of the reviewer is again faulty and as discussed above relates to disregard for genomic context. The ancestral arrangements of genes belonging to DS blocks described in *Figures 4* and *5*(with segments labeled with greek letters) were reconstructed from an analysis of the entire A. gossypii chromosome I (*Fig. 3*) and not from analyses of only genes specific to the DS blocks in question. The ancestral gene arrangements for individual DS blocks of A. gossypii chromosome I take into consideration rearrangement operations that are global. Consequently, the steps that better explain evolution of gene order in DS block 7 based on genes in chromosome I (*Fig. 5*) are quite different when only using information in DS block 7-specific genes, explaining why the ancestor is 15 steps away both from K. waltii and A. gossypii and not less. No convergent evolutionary processes are needed to explain results because optimal rearrangement scenarios occur more naturally at chromosomal and not DS block levels*.

### Comment 3. Other evidence supports the WGD hypothesis and is incompatible with PGD

Let me mention briefly two other pieces of evidence, apart from the interleaving pattern, that support the WGD hypothesis and show how they are incompatible with the PGD model.

(i) In the *S. cerevisiae *genome, 551 pairs of duplicated genes are arranged in a pattern where a series of genes in one region of the genome has a series of paralogs in the same order in another region [1,38]. Under Martin et al's PGD model, these 551 pairs originated as independent tandem duplications. Therefore, if we consider a large duplicated region such as "Block 34" which contains 13 duplicated pairs [1] in the same order on chromosomes VII and XVI, the PGD model requires that these identical orders are the result of convergent evolution. The PGD model required that after tandem duplication of each of the 13 genes, the 13 extra copies were moved to new locations and placed in the same order (and relative orientations) as their progenitors. How can that happen? Just by chance? It is extremely improbable, and the same pattern is seen in all the duplicated blocks, not just Block 34.

(ii) The conserved orientation of the blocks relative to the centromeres [1] indicates that they were formed from larger duplicated blocks (i.e. duplicated whole chromosomes) by reciprocal translocation. Under the PGD model, conservation of block orientation could only happen if a whole chromosome is subjected to a huge number of nested inversion events of the type described above, centered on one point (the centromere), so that the genes first become sorted into two monotonic subsets of the original order, and then a series of reciprocal translocations occurs. Such a model is not credible. In the last section of Discussion (titled "Gene orientation of duplicate gene components") Martin et al. discuss this issue but consider the orientations of individual genes rather than the orientations of blocks of adjacent genes. The orientation of individual genes is more liable to be affected by species-specific inversion of small segments of DNA after the WGD (e.g. the genes that are labeled in parentheses in Figure 2 of ref. [11]), and so should be less well preserved after WGD than the overall block orientation. Martin et al. state (in the same Section) that for *S. cerevisiae *homologs of genes on *A. gossypii *chromosome I, "almost half" of them show non-conserved transcriptional orientation with respect to the centromere. The relatively low level of orientation conservation of homologs of genes on *A. gossypii *chromosome I is, I believe, a special situation resulting from an unusual series of rearrangements of genes near the *MAT *locus. I find that when the whole *A. gossypii *genome is considered (520 syntenic-homolog duplicate gene pairs from Table S3 of ref. [3]), 710 of the 1040 *S. cerevisiae *genes (68%) are transcribed in the same orientation relative to the centromere as in *A. gossypii *(Additional File 4). This is significantly more than the 50% expected under the PGD model (P = 10^-17 ^by Fisher test), so falsifying a model of independent gene duplications and relocations.

#### Author's response

*Dr. Wolfe brings to discussion two additional lines of evidence that are often used in favor of the WGD hypothesis, both of which are related to the orientation (relative to the centromere) of duplicated A. gossypii-homologous genes that exist in S. cerevisiae (the 551 pairs listed in *Additional File 4*)*.

*One argument, originally presented in the comparison between the S. cerevisiae and K. lactis genomes *[1], *is that conservation of order and orientation of duplicated genes in S. cerevisiae (defining blocks when using three pre-defined criteria) can only be explained by tetraploidy and reciprocal translocations under a WGD scenario. Under this argument, alternative scenarios (e.g. the PGD hypothesis) will require that gene order and orientation of duplicates, exemplified in genes of block 34 *[1], *result from convergent evolution. However, definition of blocks, which can be arbitrary, is unnecessary under our PGD model. Syntenic gene-interleaving relationships under the PGD model are the result of genomic rearrangements that occur more parsimoniously and in concert within the larger genomic context. These rearrangements are difficult to visualize without the help of rearrangement algorithms, sometimes involve local nested inversions (*Fig. 6*), but more generally encompass a gradual rearrangement of genomic segments of different lengths within and between chromosomes. These processes do not need nested centromere-centered inversions followed by sorting in monotonic subsets, as the reviewer contends. These trivial explanations are misleading and do not portrait appropriately the complex rearrangement process. When considering gene duplications under PGD (arising by independent tandem or segmental duplications), gene orientation should not be made relative to other genes in a block because gene-interleaving patterns do not delimit the rearrangement process in a chromosome. Instead, gene orientation should be studied on a gene-by gene basis relative to the centromere and to corresponding homologues*.

*The other argument is statistical. According to the WGD hypothesis, duplicated genes in S. cerevisiae should preserve their orientation with respect to the centromere. Dr. Wolfe agrees that the low level of conservation of gene orientation of homologs of genes on A. gossypii chromosome I (only 46% of duplicated pairs preserve their orientation) does not support the WGD model, but believes this constitutes a special situation arising from the existence of the MAT locus in chromosome I. He then states that when considering all 551 duplicate pairs of the S. cerevisiae genome, 68% are transcribed in the same orientation relative to the centromere (*Additional File 4*). Because this is significantly more than the 50% expected under a pure rearrangement model (not the PGD model as Dr. Wolfe contends)(P = 10*^-17^*by Fisher test), the reviewer correctly states this falsifies a model of independent gene duplications and relocations. However, the argument is misleading because the test relates only to the validity of the WGD scenario and should involve the null hypothesis of no orientation change in gene duplicates. What the reviewer fails to mention is that because 32% of genes do change orientation and given a sample size that will make expectations highly significant, the null hypothesis of no change will be rejected by a more significant P value (P = 10*^-113^*) by Fisher test, therefore rejecting the WGD model*.

### Reviewer's report 2

Austin L Hughes, Department of Biological Sciences, University of South Carolina, Columbia, SC 29208, USA (nominated by Eugene Koonin, NCBI, NLM, NIH, Bethesda, MD 20894, USA). 29 August 2007

Ohno [37] was the original champion of the hypothesis that whole genome duplication (WGD) has played an important role in evolution. Ohno [37] had some very odd ideas about the mechanism of gene expression that led him to believe that tandem duplication could never lead to anything productive. Today we know that Ohno was wrong about tandem duplication, which in fact is a ubiquitous feature of genomes. Moreover, through functional differentiation of duplicates, tandem duplication is clearly a major source – perhaps *the *major source – of evolutionary novelty [55]. Polyploid organisms are known, but it is unclear that polyploidization has ever given rise to any important novelty. Thus, the recent obsession with alleged cases of WGD on the part of evolutionary genomicists is puzzling to say the least.

As my colleagues and I have discussed extensively elsewhere, a significant problem with virtually all published claims of WGD is that in these studies the authors show evidence of patterns consistent with WGD but they do not conduct critical hypothesis tests [56]. To my way of thinking, what is distinctive about natural science (as opposed to other forms of human intellectual effort) is the use of a specific method (the "hypothetico-deductive method") that decides among competing hypotheses by formulating falsifiable predictions of each hypothesis [57]. In science, the null or starting hypothesis must always be the hypothesis of no effect. Thus, we must always approach the study of genomic evolution with the null hypothesis that no WGD has taken place. Only if the observed results are highly unlikely under the null hypothesis should we (tentatively) accept the alternative hypothesis of WGD.

The paper of Martin et al. is unusual in that it applies a rigorous hypothesis-testing framework to WGD in the case of the yeast *S. cerevisiae*. The approach is based on the assumption that the more parsimonious evolutionary scenario is more likely. This seems reasonable, particularly since WGD advocates typically make the claim that WGD is more parsimonious than hypotheses invoking independent events of segmental duplication because the latter duplicates many genes with one event. However, Martin et al. show that in fact a hypothesis of multiple segmental duplications is more parsimonious than that of WGD, given biologically reasonable assumptions.

Of course, a limitation of any such study is that only certain scenarios are compared, since the number of theoretically possible scenarios is very large. WGD advocates will perhaps claim that there *may *exist an unexplored scenario involving WGD that would be more parsimonious than the scenarios without WGD examined here. But the burden of proof is on the WGD advocates. Unless they can present actual proof that WGD is more parsimonious, the null hypothesis stands. The paper of Martin et al. is important because it provides a solid quantitative demonstration that WGD is not a plausible hypothesis in a species for which the hypothesis of WGD has rarely if ever been questioned.

#### Author's response

*We thank Dr. Hughes for his comments and for placing our work into the right context, the Popperian hypothesis-testing framework. We apply this framework to an important question related to evolutionary change. Is genomic change gradual or saltatory? The question applies in our case to the yeast lineage and to levels of chromosomal duplication, but the theme is recurrent in biology*.

*We agree that not all possible scenarios are compared and that this limits our study. For example, our analysis proceeds by duplicating, deleting and then rearranging genes, in that order. However, there could be other more parsimonious explanations that would consider steps of duplication, deletion and rearrangement in any possible sequence. We hope a powerful algorithm and a formal distance measure can be devised in the near future that can handle this complicated and computationally intense problem*.

### Reviewer's report 3

*Mikhail S. Gelfand, Institute for Information Transmission Problems, Moscow, Russian Federation*.

The reviewer provided no comments for publication

### Reviewer's report 4

Mark Gerstein, Yale University, New Haven, CT 06520, USA)

The reviewer provided no comments for publication

## Supplementary Material

Additional file 1 Figure A. Yeast Gene Order Browser view of the region discussed in my review. Click here for file

Additional file 2 Figure B. The 19 rearrangement steps inferred in the PGD scenario in Martin et al's Figure 1B, using the GRIMM server. The yellow bars show the groups of genes moved at each step. Numbers refer to genes, numbered as in Figure 1B, except that "33" is a duplicate copy of gene 12, and "34" is a duplicate of gene 27. Negative numbers indicate genes transcribed leftwards. C35, C36, C37, C38 are chromosome segment ends ("caps"). Click here for file

Additional file 3 Figure C. Summary of the genes inverted at each step in the PGD scenario shown in Figure B. The steps consist of a series of inversions, centered on gene 12, that successively invert larger and larger regions of DNA but never spill into the neighboring region (e.g. the region to the left of gene 1). Click here for file

Additional file 4 Table A. Conservation of gene orientation between *A. gossypii *and *S. cerevisiae *gene pairs. The 520 pairs are the SH pairs from Dietrich et al. [11]. Click here for file
