# Supplementary figures and images for: Gene-interleaving patterns of synteny in the Saccharomyces cerevisiae genome: are they proof of an ancient genome duplication event?
Source: Biol Direct. 2007 Sep 25;2:23. doi: 10.1186/1745-6150-2-23 (PMC2134927; doi:10.1186/1745-6150-2-23)

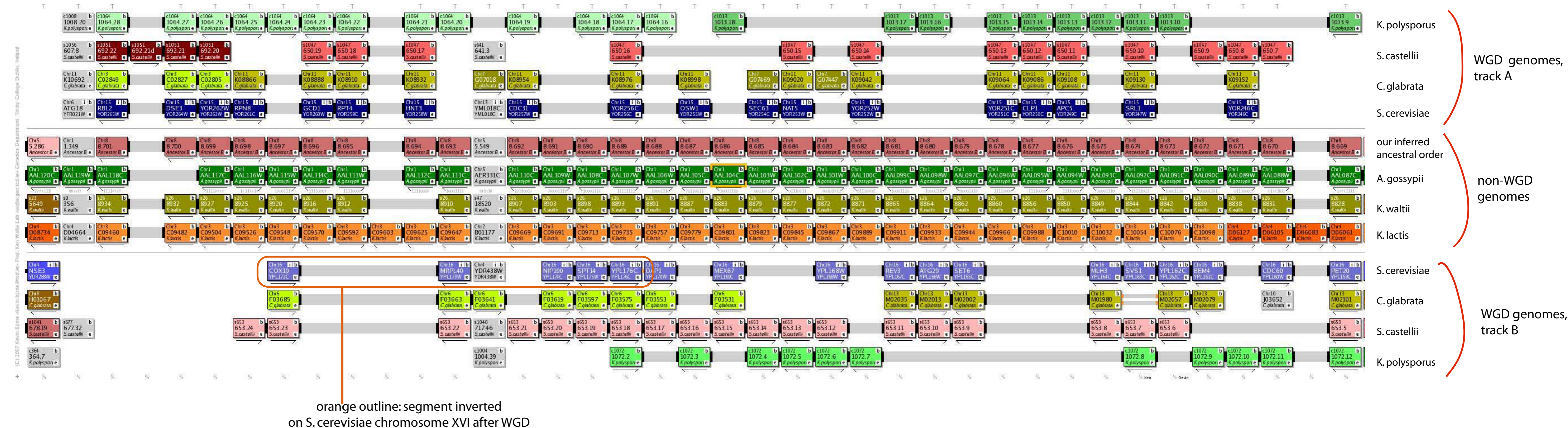

Supplement: Additional file 1 — Figure A. Yeast Gene Order Browser view of the region discussed in my review. [file 1745-6150-2-23-S1.pdf]
