# Supplementary material for: Gene-interleaving patterns of synteny in the Saccharomyces cerevisiae genome: are they proof of an ancient genome duplication event?
Source: Biol Direct. 2007 Sep 25;2:23. doi: 10.1186/1745-6150-2-23 (PMC2134927; doi:10.1186/1745-6150-2-23)
Supplement: Additional file 2 — Figure B. The 19 rearrangement steps inferred in the PGD scenario in Martin et al's Figure 1B, using the GRIMM server. The yellow bars show the groups of genes moved at each step. Numbers refer to genes, numbered as in Figure 1B, except that "33" is a duplicate copy of gene 12, and "34" is a duplicate of gene 27. Negative numbers indicate genes transcribed leftwards. C35, C36, C37, C38 are chromosome segment ends ("caps"). [file 1745-6150-2-23-S2.pdf]

Click here or scroll up to enter new data or change options.

2 chromosomes, 34 genes, 4 caps   Multichromosomal Distance: 19

One optimal rearrangement scenario

Step	Description	
0	(Source)	$-C_{36}$ $-C_{37}$ C_{35} -1 -2 3 4 -5 6 -7 -8 -9 10 -11 33 12 13 -14 -15 16 -17 18 -19 -20 21 -22 23 24 25 -26 -27 -34 -28 -29 30 31 -32 C_{38}
1	Reversal	$-C_{36}$ $-C_{37}$ C_{35} -1 -2 3 4 -5 6 -7 -8 -9 10 -11 33 -12 13 -14 -15 16 -17 18 -19 -20 21 -22 23 24 25 -26 -27 -34 -28 -29 30 31 -32 C_{38}
2	Reversal	$-C_{36}$ $-C_{37}$ C_{35} -1 -2 3 4 -5 6 -7 -8 -9 10 -11 33 -13 12 -14 -15 16 -17 18 -19 -20 21 -22 23 24 25 -26 -27 -34 -28 -29 30 31 -32 C_{38}
3	Reversal	$-C_{36}$ $-C_{37}$ C_{35} -1 -2 3 4 -5 6 -7 -8 -9 10 -11 33 14 -12 13 -15 16 -17 18 -19 -20 21 -22 23 24 25 -26 -27 -34 -28 -29 30 31 -32 C_{38}
4	Reversal	$-C_{36}$ $-C_{37}$ C_{35} -1 -2 3 4 -5 6 -7 -8 -9 10 -11 33 15 -13 12 -14 16 -17 18 -19 -20 21 -22 23 24 25 -26 -27 -34 -28 -29 30 31 -32 C_{38}
5	Reversal	$-C_{36}$ $-C_{37}$ C_{35} -1 -2 3 4 -5 6 -7 -8 -9 10 -11 33 17 -16 14 -12 13 -15 18 -19 -20 21 -22 23 24 25 -26 -27 -34 -28 -29 30 31 -32 C_{38}
6	Reversal	$-C_{36}$ $-C_{37}$ C_{35} -1 -2 3 4 -5 6 -7 -8 -9 12 -14 16 -17 -33 11 -10 13 -15 18 -19 -20 21 -22 23 24 25 -26 -27 -34 -28 -29 30 31 -32 C_{38}
7	Reversal	$-C_{36}$ $-C_{37}$ C_{35} -1 -2 3 4 -5 6 -7 -8 10 -11 33 17 -16 14 -12 9 13 -15 18 -19 -20 21 -22 23 24 25 -26 -27 -34 -28 -29 30 31 -32 C_{38}
8	Reversal	$-C_{36}$ $-C_{37}$ C_{35} -1 -2 3 4 -5 6 -7 -8 10 -11 33 -18 15 -13 -9 12 -14 16 -17 -19 -20 21 -22 23 24 25 -26 -27 -34 -28 -29 30 31 -32 C_{38}
9	Reversal	$-C_{36}$ $-C_{37}$ C_{35} -1 -2 3 4 -5 6 -7 -8 10 -11 33 19 17 -16 14 -12 9 13 -15 18 -20 21 -22 23 24 25 -26 -27 -34 -28 -29 30 31 -32 C_{38}
10	Reversal	$-C_{36}$ $-C_{37}$ C_{35} -1 -2 3 4 -5 6 -7 -9 12 -14 16 -17 -19 -33 11 -10 8 13 -15 18 -20 21 -22 23 24 25 -26 -27 -34 -28 -29 30 31 -32 C_{38}
11	Reversal	$-C_{36}$ $-C_{37}$ C_{35} -1 -2 3 4 -8 10 -11 33 19 17 -16 14 -12 9 7 -6 5 13 -15 18 -20 21 -22 23 24 25 -26 -27 -34 -28 -29 30 31 -32 C_{38}
12	Reversal	$-C_{36}$ $-C_{37}$ C_{35} -1 -2 3 -5 6 -7 -9 12 -14 16 -17 -19 -33 11 -10 8 -4 13 -15 18 -20 21 -22 23 24 25 -26 -27 -34 -28 -29 30 31 -32 C_{38}
13	Fission	$-C_{36}$ 19 17 -16 14 -12 9 7 -6 5 -3 2 1 $-C_{35}$ C_{37} -33 11 -10 8 -4 13 -15 18 -20 21 -22 23 24 25 -26 -27 -34 -28 -29 30 31 -32 C_{38}
14	Translocation	$-C_{36}$ 22 -21 20 -18 15 -13 4 -8 10 -11 33 $-C_{37}$ C_{35} -1 -2 3 -5 6 -7 -9 12 -14 16 -17 -19 23 24 25 -26 -27 -34 -28 -29 30 31 -32 C_{38}
15	Translocation	$-C_{36}$ -25 -24 -23 19 17 -16 14 -12 9 7 -6 5 -3 2 1 $-C_{35}$ C_{37} -33 11 -10 8 -4 13 -15 18 -20 21 -22 -26 -27 -34 -28 -29 30 31 -32 C_{38}
16	Translocation	$-C_{36}$ 26 22 -21 20 -18 15 -13 4 -8 10 -11 33 $-C_{37}$ C_{35} -1 -2 3 -5 6 -7 -9 12 -14 16 -17 -19 23 24 25 -27 -34 -28 -29 30 31 -32 C_{38}
17	Translocation	$-C_{36}$ 27 -25 -24 -23 19 17 -16 14 -12 9 7 -6 5 -3 2 1 $-C_{35}$ C_{37} -33 11 -10 8 -4 13 -15 18 -20 21 -22 -26 -34 -28 -29 30 31 -32 C_{38}
18	Translocation	$-C_{36}$ 29 28 34 26 22 -21 20 -18 15 -13 4 -8 10 -11 33 $-C_{37}$ C_{35} -1 -2 3 -5 6 -7 -9 12 -14 16 -17 -19 23 24 25 -27 30 31 -32 C_{38}
19	Translocation (Destination)	$-C_{36}$ -30 27 -25 -24 -23 19 17 -16 14 -12 9 7 -6 5 -3 2 1 $-C_{35}$ C_{37} -33 11 -10 8 -4 13 -15 18 -20 21 -22 -26 -34 -28 -29 31 -32 C_{38}

GRIMM 1.04 by Glenn Tesler, University of California, San Diego.
Copyright © 2001-2005, The University of California.
Contains code from GRAPPA, © 2000-2001, The University of New Mexico and The University of Texas at Austin.

Figure B. The 19 rearrangment steps inferred in the PGD scenario in Martin et al's Figure 1B, using the GRIMM server. The yellow bars show the groups of genes moved at each step. Numbers refer to genes, numbered as in Figure 1B except that "33" is a duplicate copy of gene 12, and "34" is a duplicate of gene 27. Negative numbers indicate genes transcribed leftwards. C35, C36, C37, C38 are chromosome segement ends ("caps").
