# Supplementary material for: Gene-interleaving patterns of synteny in the Saccharomyces cerevisiae genome: are they proof of an ancient genome duplication event?
Source: Biol Direct. 2007 Sep 25;2:23. doi: 10.1186/1745-6150-2-23 (PMC2134927; doi:10.1186/1745-6150-2-23)
Supplement: Additional file 3 — Figure C. Summary of the genes inverted at each step in the PGD scenario shown in Figure B. The steps consist of a series of inversions, centered on gene 12, that successively invert larger and larger regions of DNA but never spill into the neighboring region (e.g. the region to the left of gene 1). [file 1745-6150-2-23-S3.pdf]

names of genes inverted in each step

gene	1	2	3	4	5	6	7	8	9	10	11	33	12	13	14	15	16	17	18	19	20	21	22	23	24	25	26	27	34	28	29	30	31	32
**step**																																		
1													x																					
2													x	x																				
3													x	x	x																			
4													x	x	x	x																		
5													x	x	x	x	x	x																
6										x	x	x	x		x		x	x																
7									x	x	x	x	x		x		x	x																
8									x				x	x	x	x	x	x	x															
9									x				x	x	x	x	x	x	x	x														
10								x	x	x	x	x	x		x		x	x		x														
11					x	x	x	x	x	x	x	x	x		x		x	x		x														
12				x	x	x	x	x	x	x	x	x	x		x		x	x		x														
13	x	x	x		x	x	x		x				x		x		x	x		x														
14	x	x	x	x	x	x	x	x	x	x	x	x	x	x	x	x	x	x	x	x	x	x	x											
15	x	x	x	x	x	x	x	x	x	x	x	x	x	x	x	x	x	x	x	x	x	x	x	x	x	x								
16	x	x	x	x	x	x	x	x	x	x	x	x	x	x	x	x	x	x	x	x	x	x	x	x	x	x	x							
17	x	x	x	x	x	x	x	x	x	x	x	x	x	x	x	x	x	x	x	x	x	x	x	x	x	x	x	x						
18	x	x	x	x	x	x	x	x	x	x	x	x	x	x	x	x	x	x	x	x	x	x	x	x	x	x	x	x	x	x	x			
19	x	x	x	x	x	x	x	x	x	x	x	x	x	x	x	x	x	x	x	x	x	x	x	x	x	x	x	x	x	x	x	x		

Figure C. Summary of the genes inverted at each step in the PGD scenario shown in Figure B. The steps consist of a series of inversions, centered on gene 12, that successively invert larger and larger regions of DNA but never spill into the neighboring region (e.g. the region to the left of gene 1).
