# Supplementary material for: Gene-interleaving patterns of synteny in the Saccharomyces cerevisiae genome: are they proof of an ancient genome duplication event?
Source: Biol Direct. 2007 Sep 25;2:23. doi: 10.1186/1745-6150-2-23 (PMC2134927; doi:10.1186/1745-6150-2-23)
Supplement: Additional file 4 — Table A. Conservation of gene orientation between A. gossypii and S. cerevisiae gene pairs. The 520 pairs are the SH pairs from Dietrich et al. [11]. [file 1745-6150-2-23-S4.pdf]

Table A. Conservation of gene orientation between A. gossypii and S. cerevisiae gene pairs. The 520 pairs are the SH pairs from Dietrich et al.

A. gossypii	S. cer gene 1		S. cer gene2		Ag orientation	Sc1 orientation	Sc2 orientation	Sc1 conserved ?	Sc2 conserved ?
					(gene transcription towards or away from centromere)				
AAL001W	YNL001W	(DOM34)	YCL001W		towards	towards	towards	1	1
AAL018W	YNL004W	(HRB1)	YCL011C	(GBP2)	towards	towards	away	1	0
AAL022W	YLR174W	(IDP2)	YNL009W	(IDP3)	towards	away	towards	0	1
AAL028W	YLR249W	(YEF3)	YNL014W	(HEF3)	towards	away	towards	0	1
AAL029W	YLR248W	(RCK2)	YGL158W	(RCK1)	towards	away	towards	0	1
AAL040W	YDR312W	(SSF2)	YHR066W	(SSF1)	towards	away	away	0	0
AAL047C	YDR309C	(GIC2)	YHR061C	(GIC1)	away	towards	towards	0	0
AAL056C	YDR304C	(CPR5)	YHR057C	(CPR2)	away	towards	towards	0	0
AAL057C	YDR303C	(RSC3)	YHR056C	(RSC30)	away	towards	towards	0	0
AAL061C	YDR300C	(PRO1)	YHR033W		away	towards	away	0	1
AAL092C	YPL163C	(SVS1)	YOR247W	(SRL1)	away	away	away	1	1
AAL107W	YOR256C		YPL176C		towards	towards	away	1	0
AAL123W	YHR043C	(DOG2)	YHR044C	(DOG1)	towards	towards	towards	1	1
AAL127W	YKL157W	(APE2)	YHR047C	(AAP1)	towards	towards	towards	1	1
AAL130W	YJR005CX		YGR169CX		towards	towards	towards	1	1
AAL136C	YJL026W	(RNR2)	YGR180C	(RNR4)	away	towards	towards	0	0
AAL156C	YLR020C		YLL012W		away	towards	towards	0	0
AAL158W	YLL010C	(PSR1)	YLR019W	(PSR2)	towards	away	away	0	0
AAL178W	YJL079C	(PRY1)	YKR013W	(PRY2)	towards	away	away	0	0
AAL181C	YJL076W	(NET1)	YKR010C	(TOF2)	away	towards	towards	0	0
AAL185W	YML109W	(ZDS2)	YMR273C	(ZDS1)	towards	towards	towards	1	1
AAR004C	YNR001C	(CIT1)	YCR005C	(CIT2)	towards	towards	towards	1	1
AAR008W	YLR258W	(GSY2)	YFR015C	(GSY1)	away	away	towards	1	0
AAR009W	YOL016C	(CMK2)	YFR014C	(CMK1)	away	away	towards	1	0
AAR011C	YOL036W		YIR016W		towards	towards	away	1	0
AAR045C	YCR069W	(CPR4)	YNR028W	(CPR8)	towards	away	away	0	0
AAR048W	YNR026C	(SEC12)	YCR067C	(SED4)	away	towards	towards	0	0
AAR060C	YNR023W	(SNF12)	YCR052W	(RSC6)	towards	away	away	0	0
AAR065C	YNR019W	(ARE2)	YCR048W	(ARE1)	towards	away	away	0	0
AAR071W	YNR016C	(ACC1)	YMR207C	(HFA1)	away	towards	towards	0	0
AAR074C	YMR206W		YNR014W		towards	away	away	0	0
AAR082W	YPR138C	(MEP3)	YGR121C	(MEP1)	away	towards	towards	0	0
AAR086W	YGR097W	(ASK10)	YPR115W		away	away	away	1	1
AAR099W	YPR119W	(CLB2)	YGR108W	(CLB1)	away	away	away	1	1
AAR100C	YPR120C	(CLB5)	YGR109C	(CLB6)	towards	towards	towards	1	1
AAR108C	YPR132W	(RPS23B)	YGR118W	(RPS23A)	towards	away	away	0	0
AAR121W	YDR409W	(SIZ1)	YOR156C	(NFI1)	away	away	towards	1	0
AAR134W	YDR418W	(RPL12B)	YEL054C	(RPL12A)	away	away	away	1	1
AAR140W	YOR054C		YKR072C	(SIS2)	away	towards	towards	0	0
AAR146W	YLR300W	(EXG1)	YOR190W	(SPR1)	away	away	away	1	1
AAR149W	YGL054C	(ERV14)	YBR210W		away	away	away	1	1
AAR154W	YBR214W	(SDS24)	YGL056C	(SDS23)	away	away	away	1	1
AAR159C	YBR216C		YGL060W		towards	towards	towards	1	1
AAR162C	YGL062W	(PYC1)	YBR218C	(PYC2)	towards	towards	towards	1	1
AAR191C	YBR031W	(RPL4A)	YDR012W	(RPL4B)	towards	away	away	0	0
ABL005C	YLR357W	(RSC2)	YGR056W	(RSC1)	away	away	away	1	1
ABL015C	YMR233W	(TRI1)	YOR295W	(UAF30)	away	away	away	1	1
ABL018C	YOR317W	(FAA1)	YMR246W	(FAA4)	away	away	away	1	1
ABL024W	YMR102C		YKL121W		towards	towards	towards	1	1
ABL028W	YKL126W	(YPK1)	YMR104C	(YPK2)	towards	towards	towards	1	1
ABL029W	YKL127W	(PGM1)	YMR105C	(PGM2)	towards	towards	towards	1	1
ABL036C	YKL104C	(GFA1)	YMR084W		away	away	away	1	1
ABL037C	YMR086W		YKL105C		away	away	away	1	1
ABL063C	YBR078W	(ECM33)	YDR055W	(PST1)	away	away	away	1	1
ABL076W	YHR198C		YHR199C		towards	towards	towards	1	1
ABL091C	YDR025W	(RPS11A)	YBR048W	(RPS11B)	away	away	away	1	1
ABL092W	YBR049C	(REB1)	YDR026C		towards	towards	towards	1	1
ABL105W	YDR233C		YDL204W		towards	towards	towards	1	1
ABL118W	YMR285C	(NGL2)	YML118W	(NGL3)	towards	towards	towards	1	1
ABL131C	YMR275C	(BUL1)	YML111W	(BUL2)	away	towards	towards	0	0
ABL133C	YHR146W	(CRP1)	YNL173C	(MDG1)	away	away	away	1	1
ABL135C	YNL176C		YDL211C		away	away	away	1	1
ABL143C	YNL183C	(NPR1)	YDL214C	(PRR2)	away	away	away	1	1
ABL156C	YDL222C		YNL194C		away	away	away	1	1
ABL158C	YDL224C	(WHI4)	YNL197C	(WHI3)	away	away	away	1	1
ABL164C	YDL226C	(GCS1)	YNL204C	(SPS18)	away	away	away	1	1
ABL174C	YNL209W	(SSB2)	YDL229W	(SSB1)	away	towards	towards	0	0
ABL193C	YNL225C	(CNM67)	YDL239C	(ADY3)	away	away	away	1	1

ABR005C	YHL001W	(RPL14B)	YKL006W	(RPL14A)	towards	towards	towards	1	1
ABR009W	YHL003C	(LAG1)	YKL008C	(LAC1)	away	away	away	1	1
ABR020W	YMR079W	(SEC14)	YKL091C		away	away	away	1	1
ABR023C	YMR081C	(ISF1)	YKL093W	(MBR1)	towards	towards	towards	1	1
ABR033C	YGR027C	(RPS25A)	YLR333C	(RPS25B)	towards	towards	towards	1	1
ABR044C	YKL039W	(PTM1)	YHL017W		towards	towards	towards	1	1
ABR055C	YMR016C	(SOK2)	YKL043W	(PHD1)	towards	towards	towards	1	1
ABR081C	YMR031C		YKL050C		towards	towards	away	1	0
ABR089C	YKL062W	(MSN4)	YMR037C	(MSN2)	towards	towards	towards	1	1
ABR095C	YKL065C	(YET1)	YMR040W		towards	away	away	0	0
ABR098C	YMR043W	(MCM1)	YMR042W	(ARG80)	towards	away	away	0	0
ABR099C	YMR047C	(NUP116)	YKL068W	(NUP100)	towards	towards	towards	1	1
ABR111C	YPR090W		YPR089W		towards	away	away	0	0
ABR129C	YMR243C	(ZRC1)	YOR316C	(COT1)	towards	towards	towards	1	1
ABR131W	YNL121C	(TOM70)	YHR117W	(TOM71)	away	away	away	1	1
ABR144C	YMR295C		YGR273C		towards	towards	towards	1	1
ABR194C	YBR177C	(EHT1)	YPL095C		towards	towards	away	1	0
ABR215C	YER093C		YBL059W		towards	towards	towards	1	1
ABR231W	YFL042C		YLR072W		away	away	away	1	1
ABR243W	YFL048C	(EMP47)	YLR080W	(EMP46)	away	away	away	1	1
ACL032C	YIL074C	(SER33)	YER081W	(SER3)	away	away	away	1	1
ACL040C	YDL175C	(AIR2)	YIL079C	(AIR1)	away	away	away	1	1
ACL043W	YIL069C	(RPS24B)	YER074W	(RPS24A)	towards	away	away	0	0
ACL053C	YER129W	(PAK1)	YGL179C	(TOS3)	away	away	away	1	1
ACL070C	YLL045C	(RPL8B)	YHL033C	(RPL8A)	away	away	away	1	1
ACL071C	YHL034C	(SBP1)	YLL046C	(RNP1)	away	away	away	1	1
ACL072C	YLL048C	(YBT1)	YHL035C		away	away	away	1	1
ACL076W	YOR096W	(RPS7A)	YNL096C	(RPS7B)	towards	away	away	0	0
ACL080W	YNL095C		YOR092W	(ECM3)	towards	away	away	0	0
ACL084C	YOR089C	(VPS21)	YNL093W	(YPT53)	away	towards	towards	0	0
ACL105C	YHR103W	(SBE22)	YDR351W	(SBE2)	away	away	away	1	1
ACL109C	YDR353W	(TRR1)	YHR106W	(TRR2)	away	away	away	1	1
ACL131W	YLR043C	(TRX1)	YGR209C	(TRX2)	towards	towards	towards	1	1
ACL140C	YGR214W	(RPS0A)	YLR048W	(RPS0B)	away	away	away	1	1
ACL157C	YLR206W	(ENT2)	YDL161W	(ENT1)	away	away	towards	1	0
ACL173C	YBR042C		YDR018C		away	towards	towards	0	0
ACL181C	YLR342W	(FKS1)	YGR032W	(GSC2)	away	away	away	1	1
ACL184C	YLR344W	(RPL26A)	YGR034W	(RPL26B)	away	away	away	1	1
ACL189C	YGR038W	(ORM1)	YLR350W		away	away	away	1	1
ACL193C	YGR041W	(BUD9)	YLR353W	(BUD8)	away	away	away	1	1
ACL196W	YLR354C	(TAL1)	YGR043C		towards	towards	towards	1	1
ACR006C	YML002W		YML003W		towards	towards	towards	1	1
ACR012C	YEL060C	(PRB1)	YOR003W	(YSP3)	towards	away	away	0	0
ACR052W	YKL148C	(SDH1)	YJL045W		away	away	towards	1	0
ACR065C	YHR021C	(RPS27B)	YKL156W	(RPS27A)	towards	towards	towards	1	1
ACR083C	YOR042W		YDR273W	(DON1)	towards	away	away	0	0
ACR084C	YOR040W	(GLO4)	YDR272W	(GLO2)	towards	away	away	0	0
ACR098C	YPL129W	(ANC1)	YOR213C	(SAS5)	towards	towards	towards	1	1
ACR099C	YPL129W	(ANC1)	YOR213C	(SAS5)	towards	towards	towards	1	1
ACR109W	YOR222W	(ODC2)	YPL134C	(ODC1)	away	away	away	1	1
ACR112C	YPL135W	(ISU1)	YOR226C	(ISU2)	towards	towards	towards	1	1
ACR113W	YOR227W		YPL137C		away	away	away	1	1
ACR117W	YOR231W	(MKK1)	YPL140C	(MKK2)	away	away	away	1	1
ACR119W	YPL141C		YOR233W	(KIN4)	away	away	away	1	1
ACR120C	YPL143W	(RPL33A)	YOR234C	(RPL33B)	towards	towards	towards	1	1
ACR125W	YPL145C	(KES1)	YOR237W	(HES1)	away	away	away	1	1
ACR152W	YDR348C		YHR097C		away	towards	towards	0	0
ACR165W	YKL020C	(SPT23)	YIR033W	(MGA2)	away	away	away	1	1
ACR171C	YIR035C		YIR036C		towards	towards	towards	1	1
ACR190C	YBL106C	(SRO77)	YPR032W	(SRO7)	towards	away	away	0	0
ACR205W	YOR127W	(RGA1)	YDR379W	(RGA2)	away	away	away	1	1
ACR221W	YLR061W	(RPL22A)	YFL034C		away	away	away	1	1
ACR227W	YBL061C	(SKT5)	YER096W	(SHC1)	away	away	away	1	1
ACR237C	YER088C	(DOT6)	YBL054W		towards	towards	towards	1	1
ACR239C	YBL056W	(PTC3)	YER089C	(PTC2)	towards	towards	towards	1	1
ACR251C	YKR061W	(KTR2)	YJL139C	(YUR1)	towards	away	away	0	0
ACR253C	YJL138C	(TIF2)	YKR059W	(TIF1)	towards	away	away	0	0
ACR254C	YJL137C	(GLG2)	YKR058W	(GLG1)	towards	away	away	0	0
ACR255C	YJL136C	(RPS21B)	YKR057W	(RPS21A)	towards	away	away	0	0
ACR259W	YJL134W	(LCB3)	YKR053C	(YSR3)	away	towards	towards	0	0
ACR260W	YJL133W	(MRS3)	YKR052C	(MRS4)	away	towards	towards	0	0
ACR267C	YNL116W		YHR115C		towards	towards	towards	1	1
ACR278W	YOL043C	(NTG2)	YAL015C	(NTG1)	away	away	away	1	1
ACR281C	YOL045W		YAL017W	(FUN31)	towards	towards	towards	1	1
ACR282C	YAL018C		YOL047C		towards	away	away	0	0
ACR283W	YAL018C		YOL048C		away	away	away	1	1
ACR290W	YAL023C	(PMT2)	YOR321W	(PMT3)	away	away	away	1	1
ADL009W	YIL006W		YEL006W		towards	towards	towards	1	1
ADL022C	YLL021W		YLR313C		away	towards	towards	0	0
ADL036C	YLR308W	(CDA2)	YLR307W	(CDA1)	away	away	away	1	1
ADL038W	YLR310C	(CDC25	YLL016W		towards	towards	towards	1	1
ADL057W	YER070W	(RNR1)	YIL066C	(RNR3)	towards	away	away	0	0

ADL065W	YIL057C		YER067W		towards	away	away	0	0
ADL067C	YER064C		YIL056W		away	towards	towards	0	0
ADL071C	YIL053W	(RHR2)	YER062C	(HOR2)	away	towards	towards	0	0
ADL075W	YIL050W	(PCL7)	YER059W	(PCL6)	towards	towards	away	1	0
ADL077C	YIL051C	(MMF1)	YER057C	(HMF1)	away	away	towards	1	0
ADL078C	YIL052C	(RPL34B)	YER056C		away	away	towards	1	0
ADL083C	YIL045W	(PIG2)	YER054C	(GIP2)	away	towards	towards	0	0
ADL104W	YIL036W	(CST6)	YER045C	(ACA1)	towards	towards	towards	1	1
ADL127C	YFR031C		YIL018W	(RPL2B)	away	towards	towards	0	0
ADL132W	YHR149C		YGR221C	(TOS2)	towards	towards	towards	1	1
ADL140C	YGR230W	(BNS1)	YHR152W	(SPO12)	away	away	away	1	1
ADL149W	YHR158C	(KEL1)	YGR238C	(KEL2)	towards	towards	towards	1	1
ADL151W	YGR239C	(PEX21)	YHR160C	(PEX18)	towards	towards	towards	1	1
ADL157C	YOL120C	(RPL18A)	YNL301C	(RPL18B)	away	away	away	1	1
ADL158C	YNL302C	(RPS19B)	YOL121C	(RPS19A)	away	away	away	1	1
ADL168C	YNL307C	(MCK1)	YOL128C		away	away	away	1	1
ADL217W	YIL095W	(PRK1)	YNL020C	(ARK1)	towards	towards	away	1	0
ADL234C	YIL105C		YNL047C		away	away	away	1	1
ADL239C	YIL109C	(SEC24)	YNL049C	(SFB2)	away	away	away	1	1
ADL243W	YNL052W	(COX5A)	YIL111W	(COX5B)	towards	towards	towards	1	1
ADL245W	YNL053W	(MSG5)	YIL113W		towards	towards	towards	1	1
ADL247C	YNL055C	(POR1)	YIL114C	(POR2)	away	away	away	1	1
ADL251C	YNL058C		YIL117C	(PRM5)	away	away	away	1	1
ADL258W	YNL065W	(AQR1)	YIL121W		towards	towards	towards	1	1
ADL262W	YOR101W	(RAS1)	YNL098C	(RAS2)	towards	away	away	0	0
ADL273C	YOR204W	(DED1)	YPL119C	(DBP1)	away	away	away	1	1
ADL285C	YGL144C		YDL109C		away	away	away	1	1
ADL310W	YPR052C	(NHP6A)	YBR089C		towards	towards	towards	1	1
ADL318C	YBR100W		YBR098W	(MMS4)	away	away	away	1	1
ADL366W	YPR074C	(TKL1)	YBR117C	(TKL2)	towards	towards	towards	1	1
ADL370C	YPR080W	(TEF1)	YBR118W	(TEF2)	away	away	away	1	1
ADL373W	YBR121C	(GRS1)	YPR081C		towards	towards	towards	1	1
ADL384W	YHR208W	(BAT1)	YJR148W	(BAT2)	towards	away	away	0	0
ADL388W	YHR206W	(SKN7)	YJR147W	(HMS2)	towards	away	away	0	0
ADL391C	YHR203C	(RPS4B)	YJR145C	(RPS4A)	away	towards	towards	0	0
ADR026W	YAL007C	(ERP2)	YOR016C	(ERP4)	away	away	towards	1	0
ADR033W	YGR092W	(DBF2)	YPR111W	(DBF20)	away	away	away	1	1
ADR065W	YBL101C	(ECM21)	YPR030W	(CSR2)	away	away	away	1	1
ADR068W	YLR210W	(CLB4)	YDL155W	(CLB3)	away	away	towards	1	0
ADR091W	YDL194W	(SNF3)	YDL138W	(RGT2)	away	towards	towards	0	0
ADR094W	YDL192W	(ARF1)	YDL137W	(ARF2)	away	towards	towards	0	0
ADR095W	YDL191W	(RPL35A)	YDL136W	(RPL35B)	away	towards	towards	0	0
ADR099C	YDL134C	(PPH21)	YDL188C	(PPH22)	towards	away	away	0	0
ADR103C	YDL184C	(RPL41A)	YDL133C		towards	away	away	0	0
ADR107W	YDL131W	(LYS21)	YDL182W	(LYS20)	away	towards	towards	0	0
ADR109W	YDL181W	(INH1)	YDL130W		away	towards	towards	0	0
ADR119W	YBR284W		YJL070C		away	away	away	1	1
ADR126C	YKL072W	(STB6)	YMR053C	(STB2)	towards	towards	towards	1	1
ADR163W	YDR247W		YPL026C	(SKS1)	away	away	away	1	1
ADR167W	YNR047W		YCR091W	(KIN82)	away	away	away	1	1
ADR170C	YCR094W	(CDC50)	YNR048W		towards	away	away	0	0
ADR195C	YML007W	(YAP1)	YDR423C	(CAD1)	towards	towards	towards	1	1
ADR205C	YOR062C		YKR075C		towards	towards	towards	1	1
ADR209W	YOR066W		YKR077W		away	away	away	1	1
ADR211W	YOR069W	(VPS5)	YKR078W		away	away	away	1	1
ADR221C	YKR084C	(HBS1)	YOR076C	(SKI7)	towards	towards	towards	1	1
ADR231C	YKR089C		YOR081C		towards	towards	towards	1	1
ADR233W	YOR083W		YKR091W	(SRL3)	away	away	away	1	1
ADR253W	YMR139W	(RIM11)	YDL079C	(MRK1)	away	away	away	1	1
ADR257C	YDL082W	(RPL13A)	YMR142C	(RPL13B)	towards	towards	towards	1	1
ADR258W	YMR143W	(RPS16A)	YDL083C	(RPS16B)	away	away	away	1	1
ADR262C	YMR145C	(NDE1)	YDL085W	(NDE2)	towards	towards	towards	1	1
ADR272W	YMR153W	(NUP53)	YDL088C	(ASM4)	away	away	away	1	1
ADR279C	YDL095W	(PMT1)	YDL093W	(PMT5)	towards	towards	towards	1	1
ADR294C	YDR326C		YHR080C		towards	towards	towards	1	1
ADR311C	YDL022W	(GPD1)	YOL059W	(GPD2)	towards	towards	towards	1	1
ADR322W	YKR042W	(UTH1)	YJL116C	(NCA3)	away	away	away	1	1
ADR354W	YOR326W	(MYO2)	YAL029C	(MYO4)	away	away	away	1	1
ADR355C	YOR327C	(SNC2)	YAL030W	(SNC1)	towards	towards	towards	1	1
ADR366W	YOR338W		YAL034C	(FUN19)	away	away	away	1	1
ADR368W	YAL038W	(CDC19)	YOR347C	(PYK2)	away	towards	towards	0	0
ADR371W	YOR342C		YAL037W		away	towards	towards	0	0
ADR403C	YAL051W	(OAF1)	YOR363C	(PIP2)	towards	towards	towards	1	1
ADR404C	YAL051W	(OAF1)	YOR363C	(PIP2)	towards	towards	towards	1	1
ADR405C	YAL051W	(OAF1)	YOR363C	(PIP2)	towards	towards	towards	1	1
ADR407C	YAL053W		YOR365C		towards	towards	towards	1	1
ADR414C	YAL056W	(KRH1)	YOR371C	(KRH2)	towards	towards	towards	1	1
AEL013C	YDL042C	(SIR2)	YOL068C	(HST1)	away	away	away	1	1
AEL016C	YFR023W	(PES4)	YHR015W	(MIP6)	away	away	away	1	1
AEL017W	YFR024C		YHR016C	(YSC84)	towards	towards	towards	1	1
AEL027W	YOL019W	(TOS7)	YFR012W		towards	towards	away	1	0
AEL050C	YCR026C		YEL016C		away	towards	away	0	1

AEL061W	YEL022W	(GEA2)	YJR031C	(GEA1)	towards	towards	towards	1	1
AEL091C	YOL066C	(RIB2)	YDL036C		away	away	away	1	1
AEL110W	YKL164C	(PIR1)	YJL159W	(HSP150)	towards	away	towards	0	1
AEL111C	YKL163W	(PIR3)	YJL160C		away	towards	away	0	1
AEL115C	YKL166C	(TPK3)	YJL164C	(TPK1)	away	away	away	1	1
AEL118C	YKL168C	(KKQ8)	YJL165C	(HAL5)	away	away	away	1	1
AEL137W	YKL180W	(RPL17A)	YJL177W	(RPL17B)	towards	towards	towards	1	1
AEL152W	YJL191W	(RPS14B)	YCR031C	(RPS14A)	towards	towards	towards	1	1
AEL159W	YJL065C		YBR278W	(DPB3)	towards	away	away	0	0
AEL172W	YBR270C		YJL058C		towards	towards	away	1	0
AEL184W	YBR273C		YJL048C		towards	towards	away	1	0
AEL191C	YBR037C	(SCO1)	YBR024W	(SCO2)	away	towards	away	0	1
AEL201W	YNL293W	(MSB3)	YOL112W	(MSB4)	towards	towards	towards	1	1
AEL205W	YNL298W	(CLA4)	YOL113W	(SKM1)	towards	towards	towards	1	1
AEL207W	YNL299W	(TRF5)	YOL115W	(TRF4)	towards	towards	towards	1	1
AEL209W	YHR161C	(YAP1801)	YGR241C	(YAP1802)	towards	towards	towards	1	1
AEL210C	YGR243W		YHR162W		away	away	away	1	1
AEL215C	YHR163W	(SOL3)	YGR248W	(SOL4)	away	away	away	1	1
AEL222C	YOR028C	(CIN5)	YDR259C	(YAP6)	away	towards	towards	0	0
AEL233C	YOR019W		YDR475C		away	away	towards	1	0
AEL248C	YPL077C		YBR197C		away	away	towards	1	0
AEL254W	YBR191W	(RPL21A)	YPL079W	(RPL21B)	towards	away	towards	0	1
AEL255W	YBR189W	(RPS9B)	YPL081W	(RPS9A)	towards	away	towards	0	1
AEL264C	YPR172W		YLR456W		away	away	away	1	1
AEL266W	YLR457C	(NBP1)	YPR174C		towards	towards	towards	1	1
AEL283C	YLR356W		YGR049W	(SCM4)	away	away	away	1	1
AEL287C	YHR001W	(OSH7)	YKR003W	(OSH6)	away	away	away	1	1
AEL295C	YPR008W	(HAA1)	YGL166W	(CUP2)	away	away	towards	1	0
AEL298C	YPR009W	(SUT2)	YGL162W	(SUT1)	away	away	towards	1	0
AEL302W	YLR332W	(MID2)	YGR023W	(MTL1)	towards	away	away	0	0
AEL306C	YMR109W	(MYO5)	YKL129C	(MYO3)	away	away	away	1	1
AEL310C	YMR113W	(FOL3)	YKL132C	(RMA1)	away	away	away	1	1
AEL314W	YJL112W	(MDV1)	YKR036C	(CAF4)	towards	towards	towards	1	1
AEL325W	YPR102C	(RPL11A)	YGR085C	(RPL11B)	towards	towards	towards	1	1
AER001C	YBR001C	(NTH2)	YDR001C	(NTH1)	towards	towards	towards	1	1
AER006W	YBR005W		YDR003W		away	away	away	1	1
AER017C	YDL010W		YBR014C		towards	towards	towards	1	1
AER025C	YOL081W	(IRA2)	YBR140C	(IRA1)	towards	towards	towards	1	1
AER029C	YOL082W	(CVT19)	YOL083W		towards	towards	towards	1	1
AER032W	YOL086C	(ADH1)	YBR145W	(ADH5)	away	away	away	1	1
AER052W	YLR388W	(RPS29A)	YDL061C	(RPS29B)	away	away	away	1	1
AER068C	YLR399C	(BDF1)	YDL070W	(BDF2)	towards	towards	towards	1	1
AER076C	YDL075W	(RPL31A)	YLR406C	(RPL31B)	towards	towards	towards	1	1
AER090W	YLR413W		YKL187C		away	away	away	1	1
AER117W	YML056C	(IMD4)	YLR432W	(IMD3)	away	away	away	1	1
AER118C	YLR433C	(CNA1)	YML057W	(CMP2)	towards	towards	towards	1	1
AER122C	YLR437C		YML058W	(SML1)	towards	towards	towards	1	1
AER131C	YML063W	(RPS1B)	YLR441C	(RPS1A)	towards	towards	towards	1	1
AER133C	YML065W	(ORC1)	YLR442C	(SIR3)	towards	towards	towards	1	1
AER149W	YLR448W	(RPL6B)	YML073C	(RPL6A)	away	away	away	1	1
AER150W	YML074C	(FPR3)	YLR449W	(FPR4)	away	away	away	1	1
AER152W	YML075C	(HMG1)	YLR450W	(HMG2)	away	away	away	1	1
AER159C	YML081W		YJR127C	(ZMS1)	towards	towards	towards	1	1
AER164C	YJR130C	(STR2)	YML082W		towards	towards	towards	1	1
AER173C	YBR082C	(UBC4)	YDR059C	(UBC5)	towards	towards	towards	1	1
AER184W	YBL030C	(PET9)	YBR085W	(AAC3)	away	away	away	1	1
AER190W	YCR011C	(ADP1)	YOL075C		away	towards	away	0	1
AER192W	YCR010C	(ADY2)	YNR002C	(FUN34)	away	towards	towards	0	0
AER212W	YDL012C		YBR016W		away	away	away	1	1
AER228C	YOL056W	(GPM3)	YDL021W	(GPM2)	towards	towards	towards	1	1
AER232C	YHR030C		YKL161C	(SLT2)	towards	towards	away	1	0
AER243W	YHR108W	(GGA2)	YDR358W	(GGA1)	away	away	away	1	1
AER264C	YCR073C	(SSK22)	YNR031C	(SSK2)	towards	towards	towards	1	1
AER268W	YNR034W	(SOL1)	YCR073W		away	away	away	1	1
AER271W	YNR034W		YCR076WX		away	away	away	1	1
AER276C	YML100W	(TSL1)	YMR261C	(TPS3)	towards	towards	towards	1	1
AER279W	YMR264W	(CUE1)	YML101C		away	away	away	1	1
AER290C	YML106W	(URA5)	YMR271C	(URA10)	towards	towards	towards	1	1
AER294C	YHR174W	(ENO2)	YGR254W	(ENO1)	towards	away	away	0	0
AER303W	YML034W	(SRC1)	YDR458C		away	towards	towards	0	0
AER312W	YML028W	(TSA1)	YDR453C		away	towards	towards	0	0
AER314W	YML027W	(YOX1)	YDR451C	(YHP1)	away	towards	towards	0	0
AER315C	YML026C	(RPS18B)	YDR450W	(RPS18A)	towards	away	away	0	0
AER319W	YDR447C	(RPS17B)	YML024W	(RPS17A)	away	towards	towards	0	0
AER325W	YML022W	(APT1)	YDR441C	(APT2)	away	towards	towards	0	0
AER331C	YML018C		YDR438W		towards	away	away	0	0
AER334C	YML016C	(PPZ1)	YDR436W	(PPZ2)	towards	away	away	0	0
AER356C	YOR280C		YMR222C		towards	towards	towards	1	1
AER356C	YOR280C		YMR222C		towards	towards	towards	1	1
AER361C	YER027C	(GAL83)	YGL208W	(SIP2)	towards	towards	towards	1	1
AER361C	YER027C	(GAL83)	YGL208W	(SIP2)	towards	towards	towards	1	1
AER367C	YIL133C	(RPL16A)	YNL069C	(RPL16B)	towards	away	away	0	0

AER367C	YIL133C	(RPL16A)	YNL069C	(RPL16B)	towards	away	away	0	0
AER369C	YNL068C	(FKH2)	YIL131C	(FKH1)	towards	away	away	0	0
AER369C	YNL068C	(FKH2)	YIL131C	(FKH1)	towards	away	away	0	0
AER372C	YNL066W	(SUN4)	YIL123W	(SIM1)	towards	towards	towards	1	1
AER372C	YNL066W	(SUN4)	YIL123W	(SIM1)	towards	towards	towards	1	1
AER387C	YOR033C	(EXO1)	YDR263C	(DIN7)	towards	towards	towards	1	1
AER387C	YOR033C	(EXO1)	YDR263C	(DIN7)	towards	towards	towards	1	1
AER388C	YDR264C	(AKR1)	YOR034C	(AKR2)	towards	towards	towards	1	1
AER388C	YDR264C	(AKR1)	YOR034C	(AKR2)	towards	towards	towards	1	1
AER397C	YBL079W	(NUP170)	YER105C	(NUP157)	towards	towards	towards	1	1
AER401W	YDR368W	(YPR1)	YOR120W	(GCY1)	away	away	away	1	1
AER411W	YOR086C		YNL087W		away	towards	towards	0	0
AER424C	YNL079C	(TPM1)	YIL138C	(TPM2)	towards	away	away	0	0
AER431C	YIL135C		YNL074C	(MLF3)	towards	away	away	0	0
AER434C	YGL210W	(YPT32)	YER031C	(YPT31)	towards	towards	towards	1	1
AFL005W	YAR002C		YGL002W	(ERP6)	towards	towards	towards	1	1
AFL029W	YBR238C		YGL107C		towards	towards	away	1	0
AFL034W	YBR241C		YGL104C		towards	towards	away	1	0
AFL036C	YGL101W		YBR242W		away	towards	away	0	1
AFL049C	YPL177C	(CUP9)	YGL096W	(TOS8)	away	away	towards	1	0
AFL067W	YGL084C	(GUP1)	YPL189W	(GUP2)	towards	away	towards	0	1
AFL071C	YGL082W		YPL191C		away	towards	away	0	1
AFL082W	YGL076C	(RPL7A)	YPL198W	(RPL7B)	towards	away	towards	0	1
AFL087C	YGL071W	(RCS1)	YPL202C	(AFT2)	away	towards	away	0	1
AFL105C	YPL212C	(PUS1)	YGL063W	(PUS2)	away	away	towards	1	0
AFL112W	YGL133W	(ITC1)	YPL216W		towards	towards	towards	1	1
AFL115W	YPL219W	(PCL8)	YGL134W	(PCL10)	towards	towards	towards	1	1
AFL116W	YGL135W	(RPL1B)	YPL220W	(RPL1A)	towards	towards	towards	1	1
AFL120W	YGL139W		YPL221W	(BOP1)	towards	towards	towards	1	1
AFL128C	YMR177W	(MMT1)	YPL224C	(MMT2)	away	away	away	1	1
AFL134W	YPL228W	(CET1)	YMR180C	(CTL1)	towards	towards	towards	1	1
AFL135W	YMR181C		YPL229W		towards	towards	towards	1	1
AFL136W	YPL230W		YMR182C	(RGM1)	towards	towards	towards	1	1
AFL139W	YMR183C	(SSO2)	YPL232W	(SSO1)	towards	towards	towards	1	1
AFL148C	YMR186W	(HSC82)	YPL240C	(HSP82)	away	away	away	1	1
AFL161C	YPL249C	(GYP5)	YMR192W		away	away	away	1	1
AFL163C	YPL249C		YMR194W	(RPL36A)	away	away	away	1	1
AFL166C	YMR195W	(ICY1)	YPL250C	(ICY2)	away	away	away	1	1
AFL170C	YPL253C	(VIK1)	YMR198W	(CIK1)	away	away	away	1	1
AFL174C	YPL256C	(CLN2)	YMR199W	(CLN1)	away	away	away	1	1
AFL201W	YMR170C	(ALD2)	YMR169C	(ALD3)	towards	towards	towards	1	1
AFL206C	YDR341C		YHR091C	(MSR1)	away	towards	towards	0	0
AFL218C	YKR050W	(TRK2)	YJL129C	(TRK1)	away	away	away	1	1
AFL226W	YNL108C		YOR110W	(TFC7)	towards	away	away	0	0
AFL228W	YOR109W	(INP53)	YNL106C	(INP52)	towards	away	away	0	0
AFL229W	YNL104C	(LEU4)	YOR108W		towards	away	away	0	0
AFR007W	YDR069C	(DOA4)	YER144C	(UBP5)	away	towards	towards	0	0
AFR034W	YHR141C	(RPL42B)	YNL162W	(RPL42A)	away	towards	towards	0	0
AFR040W	YHR135C	(YCK1)	YNL154C	(YCK2)	away	towards	away	0	1
AFR043W	YNL156C		YHR133C		away	away	towards	1	0
AFR044C	YHR132W		YNL157W		towards	away	towards	0	1
AFR049W	YNL144C		YHR131C		away	away	towards	1	0
AFR069C	YKR034W	(DAL80)	YJL110C	(GZF3)	towards	away	away	0	0
AFR075C	YJL108C	(PRM10)	YJL107C		towards	away	away	0	0
AFR077W	YKR029C	(SET3)	YJL105W	(SET4)	away	towards	towards	0	0
AFR088W	YJL099W	(CHS6)	YKR027W		away	towards	away	0	1
AFR089W	YKR028W	(SAP190)	YJL098W	(SAP185)	away	away	towards	1	0
AFR101C	YJL084C		YKR021W		towards	away	away	0	0
AFR103W	YJL083W		YKR019C	(IRS4)	away	towards	towards	0	0
AFR108W	YGL045W		YGL046W		away	towards	towards	0	0
AFR129W	YHR183W	(GND1)	YGR256W	(GND2)	away	away	away	1	1
AFR139C	YDR389W	(SAC7)	YOR134W	(BAG7)	towards	away	away	0	0
AFR142C	YDR385W	(EFT2)	YOR133W	(EFT1)	towards	away	away	0	0
AFR159C	YDL127W	(PCL2)	YDL179W	(PCL9)	towards	towards	towards	1	1
AFR168W	YGL197W	(MDS3)	YER132C	(PMD1)	away	towards	towards	0	0
AFR175C	YER139C		YDR066C		towards	towards	towards	1	1
AFR194W	YGL189C	(RPS26A)	YER131W	(RPS26B)	away	away	away	1	1
AFR197W	YMR115W		YKL133C		away	away	away	1	1
AFR207C	YMR118C		YKL141W	(SDH3)	towards	towards	towards	1	1
AFR213C	YMR120C	(ADE17)	YLR028C	(ADE16)	towards	towards	towards	1	1
AFR214C	YMR121C	(RPL15B)	YLR029C	(RPL15A)	towards	towards	towards	1	1
AFR217W	YMR124W		YLR031W		away	away	away	1	1
AFR231W	YGL222C	(EDC1)	YER035W	(EDC2)	away	away	away	1	1
AFR234W	YGL224C	(SDT1)	YER037W	(PHM8)	away	away	away	1	1
AFR244C	YGL228W	(SHE10)	YFR039C		towards	towards	towards	1	1
AFR245W	YFR040W	(SAP155)	YGL229C	(SAP4)	away	away	away	1	1
AFR279C	YGL253W	(HXK2)	YFR053C	(HXK1)	towards	towards	towards	1	1
AFR285C	YKR094C	(RPL40B)	YIL148W	(RPL40A)	towards	towards	towards	1	1
AFR286W	YKR095W	(MLP1)	YIL149C	(MLP2)	away	away	away	1	1
AFR290W	YIL151C		YKR096W		away	away	away	1	1
AFR296C	YIL156W	(UBP7)	YKR098C	(UBP11)	towards	towards	towards	1	1
AFR300C	YKR100C		YIL158W		towards	towards	towards	1	1

AFR303W	YGR124W	(ASN2)	YPR145W	(ASN1)	away	away	away	1	1
AFR312W	YPR149W	(NCE102)	YGR131W		away	away	away	1	1
AFR320W	YGR136W	(LSB1)	YPR154W	(PIN3)	away	away	away	1	1
AFR322C	YGR138C	(TPO2)	YPR156C	(TPO3)	towards	towards	towards	1	1
AFR324W	YGR141W		YPR157W		away	away	away	1	1
AFR325W	YGR142W	(BTN2)	YPR158W		away	away	away	1	1
AFR330C	YOR312C	(RPL20B)	YMR242C	(RPL20A)	towards	towards	towards	1	1
AFR335C	YOL100W	(PKH2)	YDR490C	(PKH1)	towards	towards	towards	1	1
AFR337W	YDR492W		YOL101C		away	away	away	1	1
AFR343C	YOL103W	(ITR2)	YDR497C	(ITR1)	towards	towards	towards	1	1
AFR349W	YNL283C	(WSC2)	YOL105C	(WSC3)	away	away	away	1	1
AFR352C	YJR045C	(SSC1)	YEL030W	(ECM10)	towards	towards	towards	1	1
AFR356C	YJR047C	(ANB1)	YEL034W	(HYP2)	towards	towards	towards	1	1
AFR360W	YJR048W	(CYC1)	YEL039C	(CYC7)	away	away	away	1	1
AFR361C	YJR049C	(UTR1)	YEL041W		towards	towards	towards	1	1
AFR367W	YEL047C		YJR051W	(OSM1)	away	away	away	1	1
AFR378W	YJR054W		YML047C	(PRM6)	away	away	away	1	1
AFR413C	YHR010W	(RPL27A)	YDR471W	(RPL27B)	towards	away	away	0	0
AFR416C	YJR061W		YKL201C	(MNN4)	towards	away	away	0	0
AFR420W	YKL203C	(TOR2)	YJR066W	(TOR1)	away	away	away	1	1
AFR439C	YML125C		YML087C		towards	away	away	0	0
AFR441C	YML085C	(TUB1)	YML124C	(TUB3)	towards	away	away	0	0
AFR477C	YGL031C	(RPL24A)	YGR148C	(RPL24B)	towards	away	towards	0	1
AFR492W	YBL009W		YGL021W	(ALK1)	away	towards	towards	0	0
AFR500W	YLR324W		YGR004W		away	away	away	1	1
AFR505C	YLR327C		YGR008C	(STF2)	towards	towards	towards	1	1
AFR528W	YOR299W	(BUD7)	YMR237W		away	away	away	1	1
AFR545W	YPR159W	(KRE6)	YGR143W	(SKN1)	away	away	away	1	1
AFR569W	YOR293W	(RPS10A)	YMR230W	(RPS10B)	away	away	away	1	1
AFR585W	YLR371W	(ROM2)	YGR070W	(ROM1)	away	away	away	1	1
AFR587C	YGR071C		YLR373C	(VID22)	towards	towards	towards	1	1
AFR588W	YLR375W	(STP3)	YDL048C	(STP4)	away	away	away	1	1
AFR605C	YOL092W		YBR147W		towards	towards	away	1	0
AFR606C	YFR013W	(IOC3)	YOL017W	(ESC8)	towards	away	towards	0	1
AFR617C	YJL047C	(RTT101)	YBR259W		towards	away	away	0	0
AFR624W	YCR034W	(FEN1)	YJL196C	(ELO1)	away	away	away	1	1
AFR628C	YJL198W	(PHO90)	YCR037C	(PHO87)	towards	towards	towards	1	1
AFR651W	YHR155W		YNL257C	(SIP3)	away	away	away	1	1
AFR668W	YEL063C	(CAN1)	YNL270C	(ALP1)	away	away	away	1	1
AFR684C	YNL278W	(CAF120)	YLR187W		towards	towards	away	1	0
AFR688C	YLR185W	(RPL37A)	YDR500C	(RPL37B)	towards	away	towards	0	1
AFR689W	YLR183C	(TOS4)	YDR501W	(PLM2)	away	towards	away	0	1
AFR692C	YDR502C	(SAM2)	YLR180W	(SAM1)	towards	towards	away	1	0
AFR694W	YLR179C		YLR178C	(TFS1)	away	towards	towards	0	0
AFR695C	YLR177W		YDR505C	(PSP1)	towards	away	towards	0	1
AFR696C	YDR507C	(GIN4)	YCL024W	(KCC4)	towards	towards	towards	1	1
AFR698C	YDR508C	(GNP1)	YCL025C	(AGP1)	towards	towards	away	1	0
AFR710W	YCL035C	(GRX1)	YDR513W	(TTR1)	away	away	away	1	1
AFR712C	YCL036W	(GFD2)	YDR514C		towards	towards	towards	1	1
AFR713W	YCL037C	(SRO9)	YDR515W	(SLF1)	away	away	away	1	1
AFR716C	YCL040W	(GLK1)	YDR516C		towards	towards	towards	1	1
AFR718W	YCL043C	(PDI1)	YDR518W	(EUG1)	away	away	away	1	1
AFR723C	YCL048W		YDR522C	(SPS2)	towards	towards	towards	1	1
AFR730W	YCL050C	(APA1)	YDR530C	(APA2)	away	away	towards	1	0
AFR732C	YCL051W	(LRE1)	YDR528W	(HLR1)	towards	towards	away	1	0
AGL020W	YFL004W	(VTC2)	YPL019C	(VTC3)	towards	towards	away	1	0
AGL022W	YFL007W	(BLM3)	YFL006W		towards	towards	towards	1	1
AGL044C	YGL049C	(TIF4632)	YGR162W	(TIF4631)	away	away	away	1	1
AGL066W	YKL027W		YHR003C		towards	towards	towards	1	1
AGL082W	YKL035W	(UGP1)	YHL012W		towards	towards	towards	1	1
AGL091W	YLR228C	(ECM22)	YDR213W	(UPC2)	towards	towards	away	1	0
AGL100W	YLR233C	(EST1)	YDR206W	(EBS1)	towards	towards	away	1	0
AGL106C	YLR238W		YDR200C		away	away	towards	1	0
AGL126C	YLR168C		YDR185C		away	towards	towards	0	0
AGL137W	YLR164W		YDR178W	(SDH4)	towards	away	away	0	0
AGL197W	YDR146C	(SWI5)	YLR131C	(ACE2)	towards	towards	towards	1	1
AGL199C	YLR133W	(CKI1)	YDR147W	(EKI1)	away	away	away	1	1
AGL209W	YBR074W		YBR075W		towards	away	away	0	0
AGL244C	YPL032C	(SVL3)	YDR251W	(PAM1)	away	away	away	1	1
AGL245C	YPL037C	(EGD1)	YDR252W	(BTT1)	away	away	away	1	1
AGL246W	YDR253C	(MET32)	YPL038W	(MET31)	towards	towards	towards	1	1
AGL258C	YPL049C	(DIG1)	YDR480W	(DIG2)	away	away	away	1	1
AGL267C	YDR483W	(KRE2)	YPL053C		away	away	away	1	1
AGL282W	YER120W	(SCS2)	YBL091C		towards	away	away	0	0
AGL285C	YER119C		YBL089W		away	towards	towards	0	0
AGL288W	YBL087C	(RPL23A)	YER117W	(RPL23B)	towards	away	away	0	0
AGL293C	YER114C	(BOI2)	YBL085W	(BOI1)	away	towards	towards	0	0
AGL305W	YJR091C	(JSN1)	YPR042C	(PUF2)	towards	towards	towards	1	1
AGL310C	YPR043W	(RPL43A)	YJR094W		away	away	away	1	1
AGL320C	YBL039C	(URA7)	YJR103W	(URA8)	away	away	away	1	1
AGL342C	YMR310C		YGR283C		away	towards	towards	0	0
AGL354C	YGR279C	(SCW4)	YMR305C	(SCW10)	away	towards	towards	0	0

AGR022C	YLR328W		YGR010W		towards	away	away	0	0
AGR031W	YDR043C	(NRG1)	YBR066C	(NRG2)	away	towards	towards	0	0
AGR038C	YDR046C	(BAP3)	YBR068C	(BAP2)	towards	towards	towards	1	1
AGR039C	YDR046C	(BAP3)	YBR068C	(BAP2)	towards	towards	towards	1	1
AGR044C	YDR151C	(CTH1)	YLR136C	(TIS11)	towards	towards	towards	1	1
AGR049W	YLR110C	(CCW12)	YDR134C		away	towards	towards	0	0
AGR050W	YDR132C		YLR108C		away	towards	towards	0	0
AGR058W	YLR096W	(KIN2)	YDR122W	(KIN1)	away	away	away	1	1
AGR062C	YLR099C	(ICT1)	YDR125C	(ECM18)	towards	towards	towards	1	1
AGR085W	YLR089C		YDR111C		away	towards	towards	0	0
AGR097W	YLR083C	(EMP70)	YDR107C		away	towards	towards	0	0
AGR107C	YDR099W	(BMH2)	YER177W	(BMH1)	towards	away	away	0	0
AGR111W	YER174C	(GRX4)	YDR098C	(GRX3)	away	towards	towards	0	0
AGR117C	YER169W	(RPH1)	YDR096W	(GIS1)	towards	away	away	0	0
AGR120C	YER166W	(DNF1)	YDR093W	(DNF2)	towards	away	away	0	0
AGR138W	YDR077W	(SED1)	YER150W	(SPI1)	away	away	away	1	1
AGR154C	YER158C		YDR085C	(AFR1)	towards	towards	towards	1	1
AGR179W	YLR223C	(IFH1)	YDR223W		away	towards	away	0	1
AGR197C	YBR181C	(RPS6B)	YPL090C	(RPS6A)	towards	towards	away	1	0
AGR198C	YPL089C	(RLM1)	YBR182C	(SMP1)	towards	away	towards	0	1
AGR199W	YPL087W	(YDC1)	YBR183W	(YPC1)	away	towards	away	0	1
AGR209W	YPL105C		YBR172C	(SMY2)	away	away	towards	1	0
AGR212W	YPL106C	(SSE1)	YBR169C	(SSE2)	away	away	towards	1	0
AGR256W	YLR260W	(LCB5)	YOR171C	(LCB4)	away	away	towards	1	0
AGR261W	YLR264W	(RPS28B)	YOR167C	(RPS28A)	away	away	towards	1	0
AGR268W	YLR270W		YOR173W		away	away	away	1	1
AGR275C	YOR178C	(GAC1)	YLR273C	(PIG1)	towards	towards	towards	1	1
AGR279C	YLR277C	(YSH1)	YOR179C		towards	towards	towards	1	1
AGR283C	YOR180C	(DCI1)	YLR284C	(ECI1)	towards	towards	towards	1	1
AGR287C	YLR287C		YOR182C	(RPS30B)	towards	towards	towards	1	1
AGR294C	YOR185C	(GSP2)	YLR293C	(GSP1)	towards	towards	towards	1	1
AGR304W	YDR277C	(MTH1)	YOR047C	(STD1)	away	towards	towards	0	0
AGR306C	YJL020C	(BBC1)	YJL021C		towards	away	away	0	0
AGR312W	YJL019W		YJL018W		away	towards	towards	0	0
AGR313W	YJL017W		YJL016W		away	towards	towards	0	0
AGR315C	YGR188C	(BUB1)	YJL013C	(MAD3)	towards	towards	away	1	0
AGR316C	YJL012C	(VTC4)	YJL012C		towards	away	away	0	0
AGR344W	YNL130C	(CPT1)	YHR123W	(EPT1)	away	away	away	1	1
AGR370W	YER098W	(UBP9)	YBL067C	(UBP13)	away	away	away	1	1
AGR371C	YER099C	(PRS2)	YBL068W	(PRS4)	towards	towards	towards	1	1
AGR373C	YER101C	(AST2)	YBL069W	(AST1)	towards	towards	towards	1	1
AGR375W	YBL072C	(RPS8A)	YER102W	(RPS8B)	away	away	away	1	1
								total 365 of 520	total 345 of 520

Overall, 710 of 1040 S. cer. duplicated genes are in the same orientation relative to the centromere as their A. gossypii homologs.
